# Anaerobic hexadecane degradation by a thermophilic Hadarchaeon from Guaymas Basin

**DOI:** 10.1093/ismejo/wrad004

**Published:** 2024-01-10

**Authors:** David Benito Merino, Julius S Lipp, Guillaume Borrel, Antje Boetius, Gunter Wegener

**Affiliations:** Max Planck Institute for Marine Microbiology, Celsi­usstraße 1, 28359, Bremen, Germany; Faculty of Geosciences, University of Bremen, Klagenfurter Straße 2, 428359, Bremen, Germany; MARUM, Center for Marine Environmental Sciences, University of Bremen, Leo­bener Straße 8, 28359, Bremen, Germany; Department of Microbiology, Unit Evolutionary Biology of the Microbial Cell, Institut Pasteur, 25 rue du Dr Roux, 75015, Paris, France; Max Planck Institute for Marine Microbiology, Celsi­usstraße 1, 28359, Bremen, Germany; MARUM, Center for Marine Environmental Sciences, University of Bremen, Leo­bener Straße 8, 28359, Bremen, Germany; Alfred Wegener Institute Helmholtz Center for Polar and Marine Research, Am Handelshafen 12, 27570, Bremerhaven, Germany; Max Planck Institute for Marine Microbiology, Celsi­usstraße 1, 28359, Bremen, Germany; MARUM, Center for Marine Environmental Sciences, University of Bremen, Leo­bener Straße 8, 28359, Bremen, Germany

**Keywords:** Archaea, alkanes, hydrocarbons, anaerobic metabolism, evolution

## Abstract

*Hadarchaeota* inhabit subsurface and hydrothermally heated environments, but previous to this study, they had not been cultured. Based on metagenome-assembled genomes, most *Hadarchaeota* are heterotrophs that grow on sugars and amino acids, or oxidize carbon monoxide or reduce nitrite to ammonium. A few other metagenome-assembled genomes encode alkyl-coenzyme M reductases (Acrs), *β*-oxidation, and Wood-Ljungdahl pathways, pointing toward multicarbon alkane metabolism. To identify the organisms involved in thermophilic oil degradation, we established anaerobic sulfate-reducing hexadecane-degrading cultures from hydrothermally heated sediments of the Guaymas Basin. Cultures at 70°C were enriched in one Hadarchaeon that we propose as *Candidatus* Cerberiarchaeum oleivorans. Genomic and chemical analyses indicate that *Ca*. C. oleivorans uses an Acr to activate hexadecane to hexadecyl-coenzyme M. A *β*-oxidation pathway and a tetrahydromethanopterin methyl branch Wood–Ljungdahl (mWL) pathway allow the complete oxidation of hexadecane to CO_2_. Our results suggest a syntrophic lifestyle with sulfate reducers, as *Ca*. C. oleivorans lacks a sulfate respiration pathway. Comparative genomics show that Acr, mWL, and *β*-oxidation are restricted to one family of *Hadarchaeota*, which we propose as *Ca*. Cerberiarchaeaceae. Phylogenetic analyses further indicate that the mWL pathway is basal to all *Hadarchaeota*. By contrast, the carbon monoxide dehydrogenase/acetyl-coenzyme A synthase complex in *Ca*. Cerberiarchaeaceae was horizontally acquired from *Bathyarchaeia*. The Acr and *β*-oxidation genes of *Ca*. Cerberiarchaeaceae are highly similar to those of other alkane-oxidizing archaea such as *Ca*. Methanoliparia and *Ca*. Helarchaeales. Our results support the use of Acrs in the degradation of petroleum alkanes and suggest a role of *Hadarchaeota* in oil-rich environments.

## Introduction

The methyl-coenzyme M reductase (Mcr) is an enzyme unique to the domain *Archaea*. Originally, this enzyme was described from methanogens, strict anaerobes that form methane from various substrates such as CO_2_, formate, acetate, and methylated compounds [1]. Anaerobic methane-oxidizing archaea (ANME) use Mcr variants to activate methane to methyl-CoM [2], and they completely oxidize the carbon to CO_2_ using a reverse methanogenesis pathway [3]. Related members of the *Halobacterota*, namely *Ca*. Syntrophoarchaeum, *Ca*. Alkanophaga, *Ca*. Ethanoperedens, and *Ca*. Argoarchaeum, thrive on short- and midchain alkanes [4-7]. They activate alkanes with divergent Mcr types (alkyl-coenzyme M reductases, Acrs) and form the corresponding alkyl-CoM [4-7]. Subsequently, these anaerobic multicarbon alkane degraders (ANKA) [8, 9] convert the alkyl-CoM to acyl-CoA, which is completely oxidized to CO_2_ via the *β*-oxidation and the Wood–Ljungdahl (WL) pathways [4, 5]. In the case of *Ca*. Ethanoperedens and *Ca.* Argoarchaeum, they activate ethane to ethyl-CoM, convert it to acetyl-CoA, and oxidize it to CO_2_ via the WL pathway [6, 7]. Most ANME and other short- and midchain alkane-oxidizing archaea do not possess respiratory pathways to couple the oxidation of their substrates to sulfate reduction [4-7, 10-14]. Instead, they form syntrophic interactions with partner sulfate-reducing bacteria (SRB) of the phylum *Desulfobacterota*, such as members of the Seep-SRB, *Ca. Desulfofervidus auxilii*, or *Thermodesulfobacteria* [4-6, 15, 16]. By contrast, *Ca.* Methanoliparia are nonsyntrophic ANKA. *Ca.* Methanoliparia couple the oxidation of alkanes via Acr activation to CO_2_-reducing methanogenesis via Mcr [17-19]. *Ca.* Methanoliparia oxidizes long-chain alkanes (chain length C_≥13_) and benzene- and cyclohexane-substituted alkanes [19].

Recent metagenomic studies revealed the presence of either Mcr or Acr in metagenome-assembled genomes (MAGs) of uncultured archaea related to classical methanogens, such as *Archaeoglobi*, and also distant groups such as *Bathyarchaeia*, *Ca.* Helarchaeales, and *Hadarchaeota* [20-23]. Previous work suggests that gene duplication and horizontal gene transfer (HGT) events have been important in the evolution of Mcr/Acr genes and alkane metabolisms in archaea [24]. Yet, neither these Acr-containing organisms, nor close relatives have been cultured. Among them, *Hadarchaeota* might have a globally relevant role, because of their wide distribution in subsurface environments [25]. *Hadarchaeota* were originally described as the South African Goldmine Miscellaneous Euryarchaeal Group, which were found in alkaline sulfate-rich mining fissure waters [26]. *Hadarchaeota* inhabit diverse anoxic subsurface habitats such as hot springs, hydrothermal sediments, deep marine sediments, aquifers, and cold seeps [23, 25, 27-30]. The high GC content (57%–61%) in the sequenced rRNAs suggested that *Hadarchaeota* are thermophiles [26]. This lineage was recently proposed to be a phylum (*Hadarchaeota*) [31], including at least 30 MAGs from public repositories [23, 25, 32]. Of these, some hot-spring *Hadarchaeota* MAGs encode an Acr related to those of *Ca.* Methanoliparia, but their alkane substrates have not been characterized [23].

In this study, we targeted the cultivation of long-chain alkane degrading microorganisms from hydrothermal sediments of the Guaymas Basin, located in the Gulf of California at 2000 m depth [33]. Here, hydrocarbon-rich fluids diffuse from deeper layers toward the sediment surface, where they fuel the metabolism of diverse microbial communities [34-36]. Of these, we enriched anoxic microbes with hexadecane as substrate and analyzed the microbial community and metabolism by “omics and metabolite analysis.” We tested the hypothesis that members of the *Hadarchaeota* are involved in alkane degradation in thermophilic anoxic environments and suggest that *Hadarchaeota* acquired the necessary pathways for alkane metabolism via HGT from other archaea.

## Materials and methods

### Sediment sampling and culture setup

For this study, we retrieved sediments from Guaymas Basin during R/V *Atlantis* cruises AT37-06 (December 2016) and AT42-05 (November 2018) with the submersible *Alvin*. On cruise AT37-06, the push cores were taken during *Alvin* dive 4869 (27° 0.45′ N 111° 24.54′ W, water depth 2001 m) from a site densely covered by an orange mat of large sulfur-oxidizing *Beggiatoaceae* bacteria. Below the mat, temperatures rapidly increased and reached 85°C at 50 cm depth [37]. On cruise AT42-05, a push core was taken during *Alvin* dive 4991 (27° 0.69′ N, 111° 24.27′ W, water depth 2013 m) from a site covered with orange-white *Beggiatoaceae* mats. Temperatures at 50 cm depth reached at least 80°C. Both samples were immediately transferred to glass bottles sealed with butyl rubber stoppers, and the headspace was exchanged to argon. Bottled sediments were stored at room temperature until further processing. Anoxic sediment slurries were prepared as described before [38]. Homogenized sediment from 2 to 10 cm was mixed with synthetic sulfate-reducer medium [38, 39]. This slurry was distributed into replicate cultivation vials and further diluted reaching a final density of ~1 g sediment per 100 ml. The slurries were amended with1 ml *n*-hexadecane (99% purity, Sigma-Aldrich) as carbon and energy source. The vials were sealed with butyl rubber stoppers and pressured with 2 atm N_2_:CO_2_ (90:10). The bottles were incubated upside down to avoid chemical reactions of the alkane substrate with the rubber stopper. Samples were incubated at 37, 50, and 70°C with mild agitation (rotation 40 rpm). As marker for anaerobic alkane degradation, we followed the formation of sulfide using a colorimetric copper sulfate assay [40]. Cultures with hexadecane at 70°C (hexadecane70) were subsequently diluted (1,4 dilution steps) when sulfide concentrations reached between 5 and 10 mM.

### DNA extraction and 16S rRNA gene sequencing

DNA was extracted from early enrichments at 37, 50, and 70°C using the MO Bio PowerSoil DNA isolation kit (Qiagen). 16S rRNA gene amplicon libraries were prepared according to the Illumina 16S metagenomic sequencing library preparation protocol (support.illumina.com/documents/documentation/chemistry_documentation/16s/16s-metagenomic-library-prep-guide-15044223-b.pdf). We amplified the V3–V4 region for bacteria and the V4–V6 region for archaea (Supplementary Table S1). The 16S rRNA gene libraries were sequenced at CeBiTec (Bielefeld, Germany) on a MiSeq (Illumina; 2 × 300-bp paired-end run, 100 000 reads per library). Sequences were analyzed in R Statistical Software v 3.5.1 (R-project.org/) with DADA2 v. 1.14 [41]. DADA2 scripts used for 16S rRNA gene analysis are accessible on GitHub (github.com/dbenitom/Metagenomics_scripts/blob/main/dada2_archaea.R and github.com/dbenitom/Metagenomics_scripts/blob/main/dada2_bacteria.R).

### DNA extraction and metagenome sequencing

DNA samples from hexadecane70 cultures were extracted at three different stages for AT37-06 samples (February 2018, September 2018, and March 2020) and at one point for AT42-05 samples (June 2021). Sampling and experimental timepoints are schemed in Supplementary Fig. S1. For the sediment-free samples, 50 ml of culture was concentrated on 0.2 μm pore polycarbonate filters (Millipore, type GTTP filters) using a gentle vacuum (−40 kPa). DNA was extracted using the DNeasy PowerWater Kit (Qiagen). Metagenomes from February 2018 were sequenced at CeBiTec (Bielefeld, Germany) on a MiSeq (Illumina, 2 × 300-bp paired-end run, 2 × 10^6^ reads). Metagenomes from September 2018 were sequenced at the Marine Biological Laboratory (Woods Hole, USA) on a HiSeq (Illumina; 2 × 150-bp paired-end run, 1.5 × 10^6^ reads). Metagenomes from March 2020 to June 2021 were sequenced at the Max-Planck-Genome-Centre (Cologne, Germany) on a MiSeq (2 × 250-bp paired-end run, 4 × 10^6^ and 10 × 10^6^ reads, respectively).

### Metagenomics analyses

Primers and adapter sequences were removed from raw metagenomic reads and they were quality trimmed with BBduk within the BBtools package v. 35.68 (sourceforge.net/projects/bbmap/), with the parameters minlength = 50 mink = 6 hdist = 1 qtrim = r trimq = 20. Microbial community composition based on 16S rRNA gene abundance was calculated with phyloFlash v. 3.3b1 [42]. Quality-trimmed reads were coassembled in metagenomic contigs with SPAdes v. 3.9.0 [43] with default parameters. Quality-trimmed reads were mapped to the coassembly with Bowtie2 v. 2.3.2 [44] using the parameters --local --q. Metagenomic contigs were imported into the “omics analysis software anvi’” v. 6 [45, 46]. Gene prediction in metagenomic contigs was done with Prodigal v 2.6 [47]. Coding sequences were annotated with Prokka v 1.11, PFAMs, TIGRFAMs, COGs, KEGGs, and RNAmmer [48-53]. CXXCH motifs in putative multiheme cytochromes (MHCs) were searched with a custom script (github.com/dbenitom/Metagenomics_scripts/blob/main/CXXCH_search_anvio_import.sh). Metagenomic binning was done with maxbin v. 2.2.7 [54]. Bins obtained with maxbin were manually refined in anvi’o [45, 46] by removing contigs whose coverage did not match the overall coverage of the bins. Average nucleotide identity (ANI) between our bins and reference genomes was calculated with fastANI v. 1.31 [55]. Pyruvate-formate lyase (Pfl) and alkyl-succinate synthase (Ass) proteins were searched in the *Archaeoglobi* MAGs by aligning Pfl of *Archaeoglobus fulgidus* and Ass of *Desulfatibacillum alkenivorans* against the proteins of our Archaeoglobi MAGs using BLASTp [56]. Optimal growth temperatures in *Hadarchaeota* MAGs were predicted with the OGT_prediction tool [57].

### Synthesis of authentic hexadecyl-CoM standards

One equivalent (0.3 g) of sodium 2-mercaptoethanesulfonate (≥98% coenzyme M sodium salt, Sigma-Aldrich) was mixed with two equivalents (0.62 ml) of 1-bromohexadecane (97%, Sigma-Aldrich) in 2.4 ml basic ammonia solution (30% NH_4_OH pH ~12). The mix was incubated overnight at RT with gentle shaking in a vortex (500 rpm). The aqueous phase was transferred to a new vial and pH was adjusted to 7.0 with 37% HCl.

### Metabolite sample extractions

Thirty milliliters of culture were centrifuged at 4000 rpm for 30 min at RT, keeping both the pellet and the supernatant. The supernatant was removed and filtered onto polycarbonate filters (0.22 μm pore size, Merck Millipore) under gentle vacuum (>30 kPa). Filter pieces and pellet were transferred to bead beating tubes (Lysing Matrix E, MP Biomedicals) with 1 ml of acetonitrile:methanol:water (4,4,2, v,v,v). The tubes were vortexed for 10 min at maximum speed. Beads and debris were pelleted by centrifugation at 10000 rpm for 20 min at RT. The clear supernatant was transferred to glass vials and stored at 4°C.

### Liquid chromatography-mass spectrometry of metabolite extracts and standards

Chemical analysis of metabolite extracts and alkyl-CoM was done as described previously [5]. Culture extracts and hexadecyl-CoM standards were analyzed via high-resolution accurate-mass mass spectrometry on a Bruker maXis plus quadrupole time-of-flight mass spectrometer (Bruker Daltonics, Bremen, Germany) connected to a Thermo Dionex Ultimate 3000RS UHPLC system (Thermo Fisher Scientific, Bremen, Germany) via an electrospray ionization ion source. Sample aliquots (equivalent to 20% of total extract) were evaporated under a nitrogen stream and redissolved in 10 μl of methanol:water (1:1, v:v) before injection. Separation was done on an Acclaim C30 reversed phase column (Thermo Fisher Scientific, 3.0 × 250 mm, 3 μm particle size). The column oven was set to 40°C, and the binary pump was programmed with a flow rate of 0.35 ml/min and the following gradient of eluent A (acetonitrile:water:formic acid 5:95:0.1, v:v:v) and eluent B (2-propanol,acetonitrile,formic acid 90:10:0.1, v:v:v): 0% B at 0 min, ramp up to 100% B at 30 min, hold at 100% B until 50 min, and reequilibration at 0% B from 51 min until the end of the run at 60 min. Parameters for the electrospray ion source were set as described previously [5]. The mass spectrometer was set to acquire alternating scans of full scan and broad-band collision-induced dissociation spectra (25 eV collision energy) in a mass range of m/z 50–600 in negative ionization mode. Every analysis was mass-calibrated to reach mass accuracy of 1–3 ppm by loop injection of a calibration solution containing sodium formate cluster ions at the end of the analysis during the equilibration phase and using the high-precision calibration algorithm. Extracted ion chromatograms were generated using a mass window of 0.01 Da. Data processing was performed using the Compass Data Analysis software package version 5.0 (Bruker Daltonics, Bremen, Germany).

### Phylogenomic and phylogenetic analyses

For archaeal phylogenomics, 289 archaeal genomes were used to build a tree based on 76 archaeal marker proteins (Supplementary Table S2) [58]. To build phylogenetic trees for methyl/alkyl-coenzyme M reductase subunit A (McrA/AcrA), formylmethanofuran dehydrogenase (FwdABC), and carbon monoxide dehydrogenase (CdhABCDE), we annotated the gene sequences using PFAMs for McrA/AcrA and Fwd, and custom hidden Markov models for Cdh [59]. The amino acid sequences for each gene set were aligned and concatenated with the anvi’o software [45, 46], using muscle as alignment tool [60]. The alignments are available as Supplementary Material (keeper.mpdl.mpg.de/library/a5b76ef5-0a9f-475e-9ed1-602c9c70ba03/Benito_Merino_Hadarchaea/Extended_data_alignments). Maximum likelihood trees for all protein sets with 100 bootstraps were calculated using IQTree v. 2.0.3, using the –test option to estimate the best substitution model for each protein [61, 62]. The phylogenetic trees were visualized and edited on the Interactive Tree of Life web server [63].

## Results

### Enrichment of Hadarchaea from Guaymas Basin sediments

**Figure 1:**
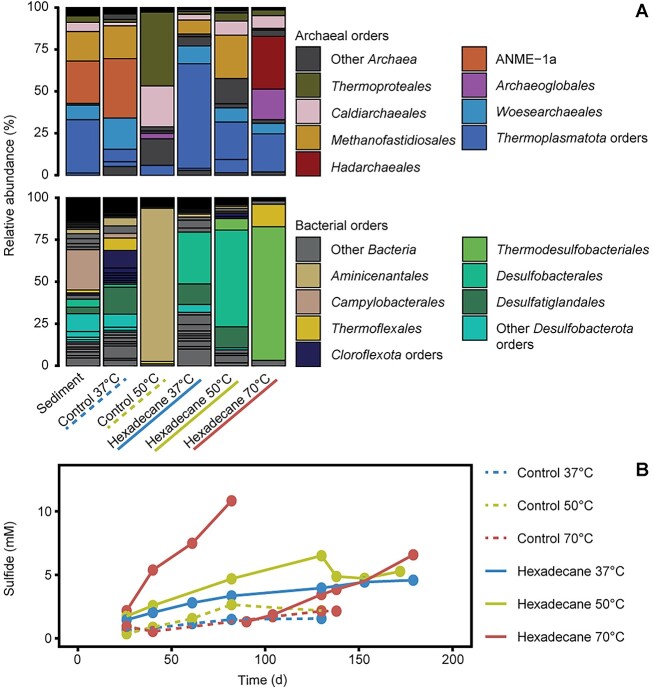
Community composition and sulfide formation in sediment slurries incubated with hexadecane; the results correspond to cruise AT37-06; (A) community composition based on archaeal and bacterial 16S rRNA gene amplicons; (B) sulfide production in early enrichments and control incubations; control incubations did not show significant sulfide production over time; enrichments with hexadecane at 37°C and 50°C showed slow activities; enrichments at 70°C grew faster and were diluted after 90 days of incubation.

We obtained sediment cores from a hydrothermally vented area in Guaymas Basin covered by sulfur-oxidizing microbial mats [37]. According to 16S rRNA gene sequencing (Fig. 1A), the main archaeal lineages in the sediment core from cruise AT37-06 are ANME-1 (~25% relative abundance of archaeal 16S rRNA gene amplicons), *Thermoplasmatota* (~30% relative abundance), *Methanofastidiosales* (~20% relative abundance), and *Woesearchaeales* (~10% relative abundance). The most abundant bacteria were SRB *Desulfobacterota* (~30% relative abundance of bacterial 16S rRNA gene amplicons) and *Campylobacterales* (~30% relative abundance).

Anoxic sediment slurries in sulfate-reducer medium were supplemented with hexadecane as sole energy source and incubated at 37, 50, and 70°C. The cultures at 37 and 50°C showed relatively slow increase of sulfide, reaching ~5 mM after 150 days (Fig. 1B). They contained mostly sulfate-reducing *Desulfobacterota* (Fig. 1A) (formerly *Deltaproteobacteria). These have been described before as alkane degraders in marine hydrocarbon seeps [64, 65]. Also, these enrichments contained large proportions of *Ca*. Thermoprofundales (phylum *Thermoplasmatota*) and *Woesearchaeales* (phylum *Nanoarchaeota*), as well as some methyl-reducing hydrogenotrophic methanogens of the order *Methanofastidiosales*, all corresponding to the groups that were present in the original sediments (Fig. 1A).

The incubation at 70°C produced 10 mM sulfide in 80 days and continued being active after a 1/5 dilution (Fig. 1B). The archaeal amplicons consisted mostly of *Hadarchaeles*, *Archaeoglobales*, and *Thermoplasmatota* (JdFR-43) (Fig. 1A). All three groups were rare or absent in the control incubations at 70°C without hexadecane, or in incubations with hexadecane at lower temperatures. *Archaeoglobales* is an order that contains free-living sulfate reducers. *A. fulgidus* was suggested to grow on hexadecane as carbon and electron source and to activate this substrate via alkylsuccinate synthases of bacterial origin [66]. The JdFR-43 family has been found in hydrothermal vents and is thought to utilize proteins and peptides for growth [67]. Also, at 70°C, most of the bacterial 16S rRNA gene amplicons were different from the lower temperature enrichments and comprised *Thermodesulfobacteriales* (Fig. 1). *Thermodesulfobacteria* are often autotrophs or grow on small organic molecules and are not known to degrade hydrocarbons [68-71]. However, they have been described recently as partners for thermophilic ANME-1c in the anaerobic oxidation of methane (AOM) and of *Ca.* Alkanophaga in the anaerobic oxidation of midchain alkanes, respectively [5, 16].

**Figure 2:**
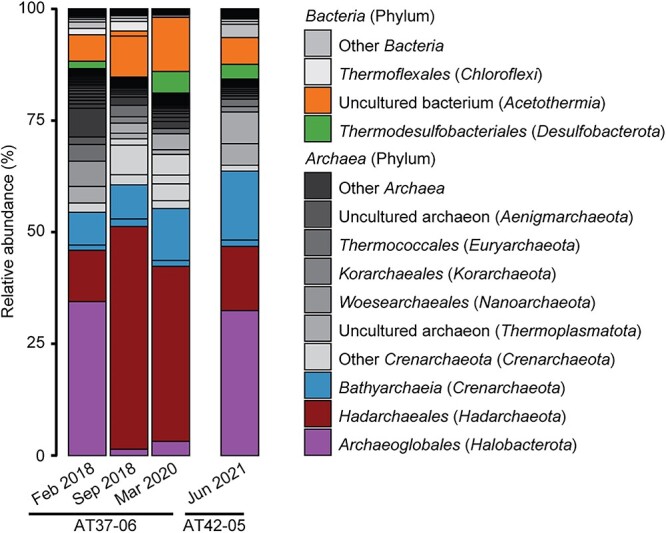
Community composition in Hexadecane70 cultures from AT37-06 to AT42-05; relative abundance of microbial taxa based on 16S rRNA gene fragments recruited from the metagenome; archaea dominate thermophilic alkane-degrading enrichments; Archaeoglobales were abundant in early sediment enrichments from the AT37-06 cruise (February 2018), and Hadarchaea became more dominant in later stages (September 2018, March 2020); the communities include heterotrophic *Bathyarchaeia* and *Acetothermia*, and sulfate-reducing *Thermodesulfobacteriales*; a second enrichment attempt from cruise AT42-05 showed similar results.

After two 1/5 dilutions, the 70°C cultures from the AT37-06 cruise were almost sediment-free. We sequenced three metagenomes at different cultivation stages to resolve its hexadecane-degrading community. Based on 16S rRNA genes recruited from the metagenomes, the cultures were dominated by *Archaea* (>85% relative abundance, Fig. 2). The early phase of the enrichments was characterized by dominance of *Archaeoglobi* (30% relative abundance), followed by *Bathyarchaeia* and *Hadarchaeota* (15%–25% relative abundance; Fig. 2). *Acetothermia* and *Thermodesulfobacteriales* sequences composed most of the bacterial fraction of the metagenome. In later culture dilutions, a shift occurred between *Archaeoglobi* and *Hadarchaeota*, with the latter becoming the most abundant group (40%–50% relative abundance), suggesting its involvement in hexadecane degradation. This enrichment consist of only two *Hadarchaeota* species as indicated by the 16S rRNA genes found in the metagenomes (Supplementary Fig. S2). We obtained similar results in an enrichment culture from a later cruise (AT42-05, Fig. 2).

From the metagenomic coassembly, we reconstructed 39 medium- to good-quality MAGs (completeness >50%, redundancy <10%, Supplementary Table S3). A *Hadarchaeota* MAG recruited ~18% of the metagenomic reads in the latest stage of the culture (91% completeness, 0% redundancy Table 1). This MAG encodes the only Acr operon present in the metagenome (Supplementary Table S4), leading to the hypothesis that these archaea may degrade hexadecane. Wang *et al*. described an Acr-encoding *Hadarchaeota* clade based on MAGs reconstructed from environmental metagenomes [23]. Here, we describe a *Hadarchaeota* MAG in our culture affiliated with this Acr-encoding *Hadarchaeota* clade. The ANI between our *Hadarchaeota* MAG and the rest of the clade is below 75% (Supplementary Fig. S3). The ANI value of our MAG is also below 75% with the placeholder genome *Ca. *Hadarchaeum yellowstonense [25, 72]. The hexadecane70 *Hadarchaeota* MAG represents a novel genus. Based on its affiliation to *Hadarchaeota* and its metabolism (see results below), we propose the species name *Ca.* Cerberiarchaeum oleivorans (see Supplementary Text).

**Table 1:** MAGs retrieved from hexadecane70 culture metagenomes (described in the main text); a complete list of MAGs can be found in Supplementary Table S3.

	Genome size (Mb)	N50 (kb)	Num. Contigs	GC content (%)	Completeness (%)	Redundancy (%)	Read recruitment Feb 2018 (%)	Read recruitment Mar 2020 (%)	Closest GTDB relative
*Candidatus* Cerberiarchaeum oleivorans	1.3 Mb	44 kb	54	52	91	0	3	18	GCA_004347925.1
*Bipolaricaulota* bacterium S4B7_HD70	1.8 Mb	51 kb	85	62	79	0	2	6	GCA_002010385.1
*Bathyarchaeia* archaeon S9B4_HD70	1.7 Mb	1.7 Mb	1	34	96	0	1	3	GCA_002254975.1
*Archaeoglobi* archaeon S5B11_HD70	0.9 Mb	15 kb	82	46	68	0	5	<1%	*A. fulgidus*
*Archaeoglobi* archaeon S5B4_HD70	2.2 Mb	27 kb	142	45	97	3	5	<1%	*A. fulgidus*

### Function of Acr in *Ca. *Cerberiarchaeum oleivorans and Acr phylogeny

**Figure 3:**
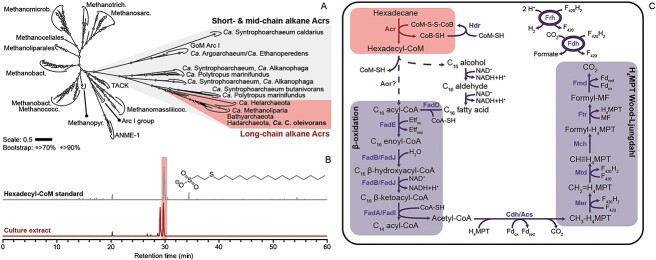
Methyl−/alkyl-coenzyme M reductase phylogeny, hexadecane activation by Acr, and proposed metabolism for *Ca*. C. oleivorans; (A) ML likelihood tree of McrA/AcrA alignment with 100 bootstraps; white circles and gray circles show bootstrap values of >70% and >90%, respectively; the clades shaded in gray include all Acr sequences (Mcr Group IV) [105]; the clade of putative long-chain alkane Acrs includes the Acr of *Ca.* C. oleivorans; (B) *Ca.* C. oleivorans activates hexadecane to hexadecyl-CoM. LC–MS analysis of hexadecane70 culture extracts shows two dominant chromatographic peaks in extracted ion chromatograms of the exact mass of hexadecyl-CoM; these peaks likely represent coenzyme M-substituted alkyls resulting from activation of the alkane in the secondary and primary position, in order of elution time [5]; (C) metabolic model for *Ca*. C. oleivorans; the Acr activates hexadecane to hexadecyl-CoM, which is then converted into a 16-carbon acyl-CoA (hexadecanoyl-CoA), possibly via Aor; acyl-CoA is processed to acetyl-CoA units (*β*-oxidation pathway); acetyl-CoA is incorporated into the downstream part of the H_4_MPT mWL via the Cdh/Acs complex; the methyl group is completely oxidized to CO_2_; the fate of the electrons released from this metabolism is unknown; F_420_H_2_ oxidation could be coupled to the production of H_2_ via Frh or to the reduction of CO_2_ to formate by an Fdh.

The genome of *Ca.* C. oleivorans harbors a single complete Acr operon. Based on the phylogenetic comparison of the catalytic alpha subunit, the Acr of *Ca.* C. oleivorans is closely related to those in MAGs of *Bathyarchaeia*, *Ca.* Helarchaeales, and *Ca.* Methanoliparia (Fig. 3A) [17-20, 22, 23]. To investigate the capacity of *Ca.* C. oleivorans to activate hexadecane with Acr, we analyzed culture extracts from the hexadecane70 culture via liquid chromatography coupled to high-resolution mass spectrometry (Supplementary Fig. S1). A peak with the exact mass of hexadecyl-CoM eluted at the same retention time as an authentic hexadecyl-CoM standard (Fig. 3B). A second peak eluted shortly before the hexadecyl-CoM standard. We hypothesize that this second compound is the product of hexadecane activation in the second carbon (2-methyl-pentadecyl-CoM, C_2_-substituted hexadecyl), as previously described for the activation of butane (C_4_ alkane) and dodecane (C_12_ alkane) [4, 5]. In *Ca.* C. oleivorans, hexadecyl-CoM is the product of the activation of *n*-hexadecane by the Acr, as described for the anaerobic short- and midchain ANKA*Ca*. Ethanoperedens, *Ca*. Syntrophoarchaeum, and *Ca*. Alkanophaga [4, 6, 7].

### Complete alkane oxidation in *Ca*. Cerberiarchaeum oleivorans 

Following Acr-dependent hexadecane activation, a conversion of hexadecyl-CoM to a hexadecanoyl-CoA (CoA-bound fatty acid) is necessary for the complete oxidation of the alkane (Fig. 3C). This mechanism is so far unknown, but for the related short-chain alkane oxidizers, candidate enzymes have been proposed based on metagenomic and metatranscriptomic data [4, 6, 7]. *Ca.* Syntrophoarchaeum butanivorans encodes a methylcobamide:CoM methyltransferase/corrinoid methyltransferase (MtaAC) that could be involved in the conversion of butyl-CoM into butyryl-CoA [4]. However, *Ca.* C. oleivorans MAG does not have the *mtaAC* genes. For *Ca.* Ethanoperedens thermophilum, a tungsten-containing aldehyde:ferredoxin oxidoreductase (Aor) has been proposed to catalyze this conversion, based on the high expression of the gene in metatranscriptomes [6]. *Ca.* C. oleivorans encodes three copies of tungsten-containing Aor (Supplementary Table S5). The function of these Aors in hexadecane degradation needs confirmation via metatranscriptomics. Due to low amounts of available culture, metatranscriptome analysis was impossible.

For *Ca.* Ethanoperedens and *Ca.* Syntrophoarchaeum, the formation of acyl-CoA from the corresponding fatty acid (acetate and butyrate) is not possible, because they do not encode acyl-CoA synthetases. By contrast, *Ca.* C. oleivorans encodes acyl-CoA synthetases (*fadD*) and alcohol dehydrogenases. Therefore, *Ca.* C. oleivorans could use long-chain fatty acids or alcohols as carbon and energy source apart from long-chain alkanes. This ability was also suggested for *Ca.* Polytropus marinifundus [21] and *Ca.* Methanoliparia [17]. Furthermore, *Ca.* C. oleivorans encodes a complete *β*-oxidation pathway, with genes present in multiple copies for several of the steps of the pathway (Supplementary Table S6). The *β*-oxidation pathway allows the production of eight acetyl-CoA units from hexadecanoyl-CoA. Most Hadarchaea (including *Ca.* C. oleivorans) encode a gluconeogenesis pathway and the C_3_-module of the glycolysis pathway for synthesis of sugars and central building blocks, respectively (Supplementary Table S7). However, none of the Hadarchaea encode a complete citric acid cycle or reductive citric acid cycle (Supplementary Table S7). In *Ca.* C. oleivorans, acetyl-CoA can be completely oxidized to CO_2_ via the CdhABCDE/acetyl-CoA synthase (Cdh/Acs) complex and the methanogenesis enzymes upstream of methyl transferase (Mtr), i.e. the tetrahydromethanopterin (H_4_MPT) methyl branch of the WL (mWL) pathway. In incubations of the enrichment cultures with ^13^C-labeled hexadecane, we measured significant production of ^13^CO_2_ over time (Supplementary Fig. S4). In total, 16 CO_2_ molecules are formed per each molecule of hexadecane, according to the following equation:


(1) \begin{equation*} {\mathrm{C}}_{16}{\mathrm{H}}_{34}+32{\mathrm{H}}_2\mathrm{O}\to 16{\mathrm{C}\mathrm{O}}_2+98{\mathrm{H}}^{+}+98{e}^{-} \end{equation*}

The liberated electrons would reduce molecules such as coenzyme F_420_, ferredoxin, NAD^+^, and flavoproteins, which need to be reoxidized in respiratory pathways, or transfer their electrons to a syntrophic partner. In our culture, alkane degradation is likely coupled to sulfate reduction. Similar to other Hadarchaea containing Acrs, *Ca.* C. oleivorans does not encode a sulfate reduction pathway. Other syntrophic ANKA produce large amounts of MHC that are likely mediating interspecies electron transfer (DIET) [4, 6, 13, 14]. *Ca. C. oleivorans* does not encode genes for MHC. Instead, it might channel the reducing equivalents in the form of molecular hydrogen produced by an F_420_-reducing NiFe-hydrogenase, or transfer small, reduced compounds like acetate and formate. Although the fermentation of hexadecane into hydrogen or acetate is unfavorable at deep-sea conditions (ΔG = 753.3 kJ or 133.9 kJ, respectively), the reactions could become feasible if syntrophic partners keep the concentrations of these compounds at low levels [73].

### Possible sulfate-reducing partners and additional associated microorganisms

We observed a good mass balance for the coupling of CO_2_ formation to sulfate reduction in the *Ca. C. oleivorans* culture (Supplementary Fig. S4A and Supplementary Text). We screened the other MAGs retrieved from the hexadecane70 culture with a focus on potential sulfate-reducing partner for *Ca.* C. oleivorans (Table 1 and Supplementary Table S3). Thermophilic ANME and their relatives *Ca.* Alkanophaga couple with *Thermodesulfobacteria* (phylum *Desulfobacterota*) to perform AOM and anaerobic oxidation of petroleum alkanes, respectively [5, 16]. *Thermodesulfobacteria* were present throughout the different stages of the hexadecane70 culture (Fig. 2). We retrieved a *Thermodesulfobacteriales* MAG in hexadecane70 metagenomes, present in low abundances (1%–2% metagenomic reads map to the MAG, Supplementary Table S3). This MAG corresponds to the species *Ca.* Thermodesulfobacterium torris, described as a partner for AOM at 70°C [16]. *Ca*. *T. torris* encodes several putative MHC that may be involved in DIET with *Ca.* C. oleivorans, alternatively to our first hypothesis of H_2_ transfer. Despite their low abundances in the total community metagenome, *Thermodesulfobacteria* are a likely partner SRB for hexadecane degradation at 70°C. In the cultures, we visualized microbial aggregates containing *Hadarchaeaota* cells (Supplementary Fig. S5). This suggests that *Ca.* C. oleivorans relies on DIET or transfer of small molecules to a syntrophic partner for the degradation of hexadecane.

In early enrichments, *Archaeoglobi* were highly abundant (Figs 1 and 2) and several MAGs recruit between 1% and 10% of metagenomic reads (Table 1 and Supplementary Table S3). The *Archaeoglobi* MAGs S5B4_HD70 and S5B11_HD70 encode a complete dissimilatory sulfate reduction pathway (Table S5) and are related to *A. fulgidus* (Supplementary Figs S6 and S7). The cultured species of the genus *Archaeoglobus* are heterotrophic or chemolithotrophic sulfate reducers [74-83]. Other *Archaeoglobi* genera (namely *Ferroglobus*, *Geoglobus* and *Ca.* Polytropus) are nitrate and ferric iron reducers [21, 84-86]. We considered whether *Archaeoglobi* from the hexadecane70 culture could receive reducing equivalents from *Ca.* C. oleivorans. However, the *Archaeoglobi* do not encode hydrogenases, making interspecies hydrogen exchange with *Ca.* C. oleivorans unlikely. They also do not code for putative MHC. We thus suggest that *Archaeoglobi* in our culture could be competitive hexadecane oxidizers using alkylsuccinate synthases (Ass), a bacterial mechanism for activation of alkanes [87]. *A. fulgidus* has been isolated from hydrothermal vents and oil reservoirs [88] and encodes a pyruvate formate lyase (Pfl) with high similarity to alkylsuccinate synthase A (AssA) and benzylsuccinate synthase A (BssA) [66]. All *Archaeoglobi* MAGs retrieved encode proteins with high similarity to Pfl/Ass that were highly expressed in *A. fulgidus* during growth on hexadecane ([66], Supplementary Fig. S8). Interestingly, one *Archaeoglobus* MAG (*Archaeoglobi* archaeon S5B9_HD70, 83% completeness, 4% contamination, Supplementary Table S3) encodes both a PflC/AssD and PflD/AssA with high sequence similarity to those of *A. fulgidus* and *D. alkenivorans*. This is the only MAG in the hexadecane70 cultures with the capacity to couple alkane oxidation to sulfate reduction within one cell (Supplementary Table S4). However, the abundance of the *Archaeoglobi* archaeon S5B9_HD70 MAG is below 0.1% in the later stages of cultivation (Supplementary Table S3). In summary, the *Archaeoglobi* of the hexadecane70 culture are unlikely to play a role as partners of *Ca.* C. oleivorans. Instead, they might compete for the oxidation of the hexadecane coupled to sulfate reduction, especially at the beginning of the cultivation.

The culture also contains a bacterial MAG affiliated to the phylum *Bipolaricaulota* (formerly *Acetothermia) that recruited 2%–6% of the metagenomic reads (Table 1). *Bipolaricaulota* have been found in anoxic environments such as oil reservoirs and anaerobic digesters [89, 90]. These bacteria are described as generalists that ferment sugars, amino acids, and peptides to acetate, formate, and hydrogen [89, 91], but do not encode MHC. In the culture, we also found a *Bathyarchaeia* MAG (phylum *Thermoproteota*) that recruited 1%–3% of the metagenomic reads (Table 1). *Bathyarchaeia* are found in diverse environments such as deep-sea and freshwater sediments [92-95]. Evans *et al*. described an environmental *Bathyarchaeia* MAG that encoded an Acr [20]. To our knowledge, no other Acr-encoding *Bathyarchaeia* have been found since then. The *Bathyarchaeia* MAG present in our culture is unlikely to be involved directly in hexadecane oxidation. Furthermore, a MAG belonging to *Ca.* Thermoprofundales (completeness <70%) recruits 1%–2% of metagenomic reads (Supplementary Table S3). *Ca.* Thermoprofundales are peptidolytic organisms that have been reported from hydrothermal environments and oil reservoirs and also co-occurring with Hadarchaea [67]. The *Bipolaricaulota*, *Bathyarchaeia*, and *Ca.* Thermoprofundales MAGs described here appear to be heterotrophic generalists that might thrive on side metabolites of the alkane-oxidizing community such as peptides, acetate, or formate [96, 97]. None of these MAGs encode for sulfate reduction genes, nor have they been previously reported as partners for AOM or anaerobic oxidation of alkanes. Overall, this community gives us insights on the complex metabolic networks that potentially operate in oil-rich environments.

**Figure 4:**
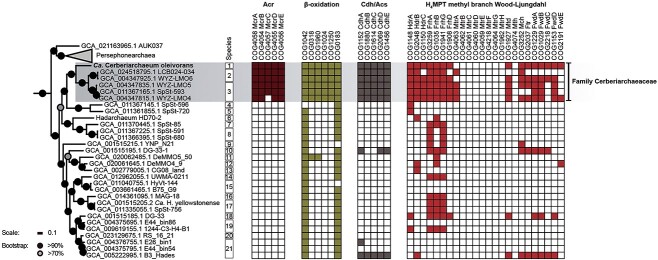
Pathways required for Acr-dependent alkane oxidation in *Hadarchaeota*; subset of a phylogenomic tree of archaea showing *Hadarchaeota* (including Persephonarchaea), and occurrence of pathways for alkane degradation in the class *Hadarchaeia*; the 95% threshold in ANI defines the 21 species of *Hadarchaeota*; colored squares indicate that the protein is encoded in the MAG; the *Ca.* Cerberiarchaeaceae family (shaded in the tree) contains MAGs encoding an Acr, a complete *β*-oxidation pathway, a Cdh/Acs, and a mWL pathway without methyl-H_4_MPT:CoM methyltransferase (Mtr); the COGs in the *β*-oxidation pathway correspond to NDP-forming acyl-CoA synthetase (COG1042), AMP-forming acyl-CoA synthetase (COG0318), acyl-CoA dehydrogenase (COG1960), enoyl-CoA hydratase (COG1024), 3-hydroxyacyl-CoA dehydrogenase (COG1250), and acetyl-CoA acetyltransferase (COG0183).

### Origin of alkane metabolism in Hadarchaea

**Figure 5:**
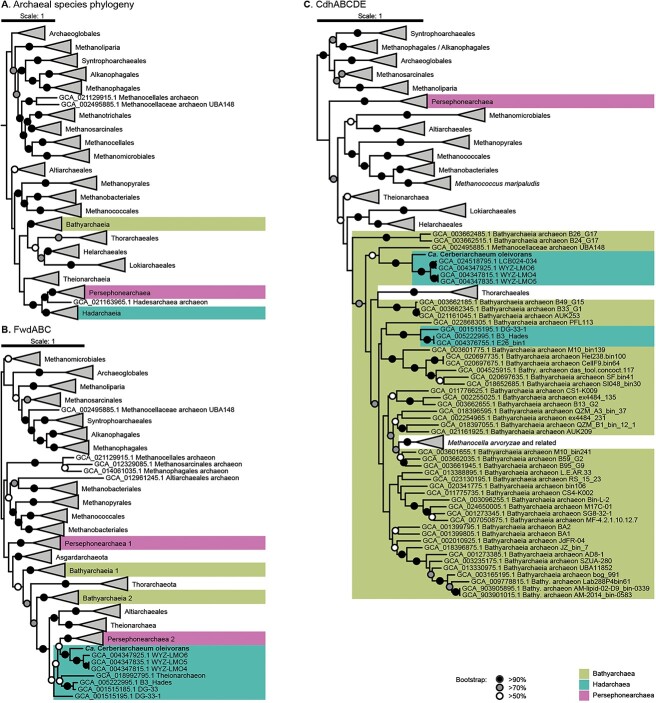
Placement of Hadarchaea, Persephonarchaea, and Bathyarchaea in species genome tree, FwdABC phylogeny, and CdhABCDE/acetyl-CoA synthase complex (Cdh) phylogeny; maximum-likelihood phylogenetic trees with 100 bootstraps based on concatenated alignment of 38 archaeal marker genes, FwdABC, and CdhABCDE protein sequences; (A) Hadarchaea and Persephonarchaea form a clade next to Theionarchaea; (B) Hadarchaea Fwd sequences form a branch with the Persephonarchaea 2 sequences; (C) Cdh sequences from the alkane-oxidizing Hadarchaea clade cluster together and branch from Bathyarchaea sequences, probably as a consequence of an event of lateral gene transfer between subsurface alkane-oxidizing archaea.

To investigate the evolutionary history of Acr-based alkane metabolism in the *Hadarchaeota*, we first determined the distribution of Acr, *β*-oxidation, and WL pathway genes in this class (Fig. 4). As previously mentioned, these pathways are necessary for short- and midchain alkane-oxidation in *Ca.* Syntrophoarchaeum and *Ca.* Alkanophaga [4, 5], and long-chain alkane oxidation coupled to methanogenesis in *Ca.* Methanoliparia [17-19]. All Hadarchaea MAGs coding for an Acr are gathered in a single family (Fig. 4) corresponding to WYZ-LMO6 in GTDB [31], for which we propose the name *Ca.* Cerberiarchaeaceae. All MAGs from this family have a complete or almost complete pathway for alkane oxidation, including genes for *β*-oxidation, both branches of the WL pathway and HdrABC genes for the regeneration of CoM-CoB. Only two other *Hadarchaeota* outside of *Ca.* Cerbariarchaeaceae encode an almost complete WL pathway. To determine the origin of alkane oxidation in the Hadarchaea, we built a reference phylogeny of archaea and compared it to the phylogeny of enzymes of the two branches of the WL pathway (Fig. 5). In the archaeal phylogeny, Persephonarchaea and Hadarchaea form a monophyletic clade, branching next to Theionarchaea (Fig. 5). The Persephonarchaea (formerly candidate division MSBL1) [98] are an uncultured group described from hypersaline anoxic basins [99]. All the Persephonarchaea MAGs have completion values of <50%. Based on our GTDB taxonomy analysis, Persephonarchaea is a sister group to the Hadarchaea, comprised within the phylum *Hadarchaeaota* and the class *Hadarchaeia* (Supplementary Table S2). Therefore, we use the term “Hadarchaea” to refer to the order *Hadarchaeales* excluding the Persephonarchaea. The Theionarchaea were described from estuary sediments and are affiliated with the *Thermococci* (Supplementary Table S2) [98, 100]. The phylogenies of the enzymes of the mWL pathway, i.e. FwdABC (Fig. 5B), formylmethanofuran—H_4_MPT N-formyltransferase (Ftr; Supplementary Fig. S9), and methenyl-H_4_MPT cyclohydrolase (Mch; Supplementary Fig. S9), are mostly congruent with the reference phylogeny of *Archaea* (Fig. 5A), supporting the results of previous phylogenies [101]. In particular, the Hadarchaea, Persephonarchaea, and Theinoarchaea clades are closely related and form a separate clade from the *Bathyarchaeia* and *Asgardarchaeota* in Fwd and Mch phylogenies, similarly to the reference tree (Fig. 5 and Supplementary Fig. S9). Altogether, this indicates that the mWL pathway was likely vertically inherited in the *Hadarchaeota*. By contrast, for the carbonyl branch of the WL pathway (i.e. ml CdhABCDE), *Ca.* Cerbariarchaeaceae sequences are distantly related to those of Persephonarchaea and Theinoarchaea and branch within the *Bathyarchaeia* (Fig. 5C). Very similar results were obtained for phylogenies based on individual Fwd and Cdh subunits (Figs S10 and S11). Therefore, Hadarchaea most likely acquired the carbonyl branch of WL by HGT from *Bathyarchaeia*. The Acr from *Hadarchaeota* are closely related to sequences from two other groups, *Bathyarchaeia* and *Halobacterota* (*Ca.* Methanoliparia), suggesting HGT between these lineages, but it is not possible to conclude on the direction of the transfer. Based on BLASTp comparisons, the *β*-oxidation genes of *Ca. C. oleivorans* are also related to those of putative long-chain alkane oxidizers from phylogenetically distant lineages, such as *Bathyarchaeia* BA1/BA2 and *Ca.* Methanoliparia (Supplementary Table S6) [17, 20]. Similarly to Acr, this supports the existence of HGT between these lineages.

## Discussion

Before this study, *Hadarchaeota* was a phylum described exclusively from environmental MAGs, and no physiological studies were available to link their genomic potential with their ecological niches. Based on environmental MAGs and 16S rRNA sequences, Hadarchaea are present in a broad range of subsurface anoxic environments, and they are associated with methane seeps and oil-rich environments (Supplementary Fig. S12). The presence of genes for CO and H_2_ metabolism supports their competitiveness in such environments [25, 102]. Furthermore, Hadarchaea are likely thermophiles, suggested, for example, by the extremely high frequency of G-quadruplex-prone regions in their DNA [103]. Predictions on optimal growth temperatures based on Hadarchaea MAGs showed that they are adapted to temperatures between 50 and 82°C (Supplementary Table S7). Most Hadarchaea lack the WL pathway (Fig. 4). Although *Ca.* Cerberiarchaeaceae might use the WL pathway for carbon assimilation, other Hadarchaea might assimilate carbon via the reductive pentose phosphate cycle (Supplementary Table S7). Subsurface Hadarchaea might couple the oxidation of carbon monoxide to the reduction of H_2_O to hydrogen or to dissimilatory nitrite reduction to ammonia, as proposed for Hadarchaea MAGs from Yellowstone [25]. The predominant metabolism in deep anoxic and oil-rich environments might be fermentation of organic compounds, rather than respiration [73]. The metabolism of non-ANKA Hadarchaea remains poorly understood without cultured representatives and further environmental mapping. The results of our enrichment study, together with other environmental data [23], now explain their presence in oil seeps and reservoirs. The genomic data along with the analysis of metabolites suggest that *Ca.* C. oleivorans uses an Acr to activate hexadecane to hexadecyl-CoM and can potentially oxidize the alkane completely to CO_2_, supporting the proposed hypothesis for Acr-based alkane oxidation in Hadarchaea [23].

This study of an enrichment culture of *Ca.* C. oleivorans suggests that it does not encode respiratory pathways or other electron sinking mechanisms. Syntrophic interactions based on transfer of molecular hydrogen or formate have been proposed [104]. The most likely partner SRB in our culture is a *Thermodesulfobacterium*, as previously proposed for AOM and midchain alkane oxidation at 70°C [5, 16]. Further cultivation efforts are needed to decipher this potential interaction between Hadarchaea and *Thermodesulfobacteria* aided by metatranscriptomics, physiological experiments, and microscopy.

In the Mcr/Acr phylogeny, we can distinguish four groups [8, 105]. Group I contains Mcrs from CO_2_-reducing methanogens and Group II corresponds to methyl-reducing methanogens. Groups I and II also contain Mcrs involved in AOM. Group III contains TACK-like Mcr sequences from *Ca*. Verstraetearchaeota, *Ca*. Nezharchaeota, *Ca*. Korarchaeota, Thaumarchaeota and Archaeoglobi [23, 106-109]. Group IV is a monophyletic clade including all Acrs. As the Acr of *Ca.* Polytropus marinifundus is closely relate to those of short- and medium-chain alkane-oxidizing archaea [4-7], we can infer that *Ca.* Polytropus marinifundus uses its Acr to activate alkanes within the range C_3_–C_14_, based on Acr phylogeny (Fig. 3A) [21]. Based on the results of Zhou *et al*. [19] and our hexadecane70 enrichments, Acrs from the Hadarchaea/*Ca.* Methanoliparia clade are all likely responsible for the activation of long-chain alkanes (Fig. 3A). Whether long-chain alkane Acrs are a monophyletic group should be investigated following cultivation of *Bathyarchaeia* and *Ca.* Helarchaeales from hydrocarbon-rich environments [20, 22].

We investigated the occurrence of pathways for Acr-based long-chain alkane oxidation in other *Hadarchaeota* MAGs available in public databases. Neither the WL pathway, nor the *β*-oxidation pathway are present in *Hadarchaeota* genomes outside of the *Ca.* Cerberiarchaeaceae, with the exception of two MAGs that encode an almost complete WL pathway (DG-33-1 and B3_Hades, Fig. 4). In absence of the *β*-oxidation pathway and Acr, these two organisms might use this WL pathway for CO_2_ fixation.

Previous studies suggested that Acr-based alkane-oxidation was transferred multiple times via HGTs in *Archaea*, but the direction of these transfers could not be resolved for most of the enzymes [24]. However, we found that *Ca.* Cerberiarchaeaceae likely acquired the carbonyl branch of the WL pathway through HGT from Bathyarchaeota, similarly to what had already been proposed for *Methanocella arvoryzae* [59]. Because this step is likely mandatory for the Acr-based alkane-oxidation, this transfer indicates that the last common ancestor of *Hadarchaeota* was likely not an ANKA and that this metabolism was acquired by HGT, at the base of the *Ca.* Cerberiarchaeaceae. In this context, other steps of the Acr-based alkane-oxidation could have been gained by HGT in *Ca.* Cerberiarchaeaceae, and in particular Acr and the *β*-oxidation pathway. These genes might have been horizontally acquired from *Bathyarchaeia* and/or *Ca.* Methanoliparia also dwelling in subsurface hydrothermal and oil-rich environments. By contrast, the mWL was vertically inherited from the LACA to *Hadarchaeaota* but was lost in members of the phylum lacking the pathway.

Overall, our study highlights the need to sample new locations and to use cultivation-based approaches to understand the extension, evolution, and physiology of Acr-based alkane metabolism in Hadarchaea and other archaea from extreme environments.

## Supplementary Material

20230915_Supplementary_Information_wrad004

## Data Availability

The 16S rRNA gene amplicons reads, raw metagenomic reads, metagenomic assembly and MAGs generated in this study are accessible under BioProject PRJNA891685.
