# Supplementary material for: Anaerobic hexadecane degradation by a thermophilic Hadarchaeon from Guaymas Basin
Source: ISME J. 2024 Jan 10;18(1):wrad004. doi: 10.1093/ismejo/wrad004 (PMC10811742; doi:10.1093/ismejo/wrad004)
Supplement: 20230915_Supplementary_Information_wrad004 [file 20230915_supplementary_information_wrad004.pdf]

Anaerobic hexadecane degradation by a thermophilic Hadarchaeon from Guaymas Basin

Description of proposed species *Candidatus* Cerberiarchaeum oleivorans and family *Ca*. Cerberiarchaeaceae

Candidatus Cerberiarchaeum: Cer.be.ri.ar.chae'um. N.L. neut. n. *archaeum*, archaeon, from Gr. adj. archaios, ancient; N.L. neut. N. *Cerberiarchaeum*, archaeon named after Cerberus (gen. *Cerberi*), the three-headed hound that guards the gates of the Underworld in the Greek mythology, in relation with the affiliation of this organism with Hadarchaea. *Candidatus* Cerberiarchaeum oleivorans: o.le.i.vo'rans. N.L. neut. n. *oleum*, oil (gen. *olei*), and *vorans*, N.L. v., eating, devouring, oil-eating *Cerberiarchaeum*.

Family Cerberiarchaeaceae: Cer.be.ri.ar.chae.a'ce.ae. N.L. neut. N. *Cerberiarchaeum* and –*aceae*, a suffix for family; N.L. fem. pl. *Cerberiarchaeaceae*, the Cerberiarchaeum family.

16S rRNA-targeted probe design

Hadarchaeota-specific probes were designed in ARB [1]. 16S rRNA gene sequences from *Hadarchaeota* MAGs were aligned to the SILVA SSU reference database (release 138.1) [2] using the SINA aligner [3]. We designed probes matching the *Ca*. Cerberiarchaeum oleivorans 16S rRNA sequence and we tested the coverage against Hadarchaea 16S rRNA sequences. The probe Hades302 (Table S1) with a coverage of 61% of the *Hadarchaeota* phylum was used for in situ hybridization.

CARD-FISH and microscopy analysis

We fixed 5 mL culture samples in 1% formaldehyde at 4°C for 2 h and washed twice in 1×PBS. We stored fixed samples in 1×PBS:ethanol (1:1, v/v) at –20°C until further processing. 500 µL of fixed sample were filtered onto GTTP polycarbonate filters (0.2 µm pore size; Millipore). Samples were embedded with 0.2% low-melting agarose and

permeabilized with lysozyme (10 mg/mL lysozyme in 0.05 M EDTA, 0.1 M Tris-HCl, pH 7.4; 1 h at 37°C), proteinase K (150 µg/mL proteinase K in 0.05 EDTA, 0.1 M Tris-HCl, 0.5 M NaCl; 10 min at RT) and HCl (0.1 M HCl; 5 min at RT). Endogenous peroxidases were inactivated with 0.15% H_2O_2 in methanol (30 min at RT). Probe solution (50 ng/µL) was diluted 1/300 in hybridization buffer (10% formamide, 0.45 M NaCl, 0.02 M Tris-HCl, 0.01% SDS, pH 7.4). Hybridization was performed for 4 h at 46°C. Signal amplification was done with Alexa Fluor 488-labelled tyramides (2 h at 46°C). Filters were mounted on microscope slides with mounting medium containing DAPI (1/1000 DAPI in citifluor:vectashield 1:1). Images were taken in a Zeiss epifluorescence microscope (Axiophot II equipped with an AxioCamMR camera) using the AxioVision software.

^{13}C-hexadecane consumption experiment

^{13}C-labeled hexadecane (hexadecane-1,2-$^{13}C_2$, 99 atom % ^{13}C, Sigma-Aldrich) was mixed 1:9 (v:v) with unlabeled hexadecane. For replicate experiments, 40 mL culture were diluted in 50 mL fresh sulfate-reducer medium (containing 10 mM sulfate) and amended with 200 µL of the $^{13}C/^{12}C$-hexadecane mix. We monitored replicate cultures for dissolved inorganic carbon (DIC), headspace CO_2 and sulfide/sulfate production. For headspace samples, 1 mL headspace was transferred to gas-tight 12-mL Exetainer vials previously gassed with synthetic air. For DIC samples, 1.5 mL of culture were sterile filtered (0.2 µm pore size GTTP syringe filter, Millipore) to completely fill 1.5-mL glass LC vials (Zinsser). For sulfide/sulfate samples, 1 mL of culture was sterile filtered and added to 0.5 mL of 100 mM zinc acetate solution. All samples were stored at 4°C until further processing. To convert DIC into CO_2 prior to analysis, 1 mL of DIC samples were transferred to gas-tight vials containing 100 µL 45% phosphoric acid solution and incubated overnight at RT. DIC/headspace samples were analyzed by isotope ratio infrared spectroscopy (Thermo Fisher, Delta Ray IRIS with URI connect and Cetac ASX-7100 autosampler). For sulfide/sulfate samples, 1 m of filtrate was added to 0.5 mL 100 mM zinc acetate solution. Samples were stored at 4°C until analysis. Sulfate/sulfide concentrations were measured by ion chromatography (930 compact IC, Metrohm). The overall isotopic composition of hexadecane in the incubations was

measured by gas chromatography (Trace GC Ultra; Thermo Fisher) coupled via a combustion interface (GC combustion III; Thermo Finnigan) to an Delta Plus XP isotope ratio mass spectrometer (Thermo Finnigan) .

Calculation of electron balance between hexadecane oxidation and sulfate reduction

We calculated the electron balance between sulfide production and DIC production in an 1,2-^{13}C-hexadecane labeling experiment. We derived a stoichiometry of sulfate reduction to DIC production according to equation 2. These calculations based on the incubation period between days 35 to 69

$$C_{16}H_{34}+12.25\,SO_4^{2-}+8.5\,H^{+}\rightarrow 16\,HCO_3^{-}+12.25\,H_2S+H_2O \qquad \text{(eq. 2)}$$

To calculate how much labeled hexadecane was oxidized to DIC we used the equation:

$$\delta\text{-}DIC_{T_0}\times C_{T_0}+\delta\text{-}DIC_{substrate}\times C_{substrate}=\delta\text{-}DIC_{T_f}\times C_{T_f}=\delta\text{-}DIC_{T_f}\times\left(C_{T_0}+C_{substrate}\right) \text{ (eq. 3),}$$

where $\delta\text{-}DIC_{T_0}$ is the ^{13}C enrichment (‰) in the DIC at day 35, C_{T_0} is the total DIC concentration (mM) at day 35, $\delta\text{-}DIC_{substrate}$ is the ^{13}C enrichment (‰) in the hexadecane, $C_{substrate}$ is the concentration of DIC (mM) produced from hexadecane oxidation during the incubation, $\delta\text{-}DIC_{T_f}$ is the ^{13}C enrichment (‰) in the DIC at day 69, and C_{T_f} is the total DIC concentration (mM) at day 69.

Based on the concentration development of total DIC ($^{13+12}$C-DIC) measured we calculated a DIC production of 2.26 mM (replicate 1) and 2.96 mM (replicate 2) (Figure S4A). According to equation 2, the sulfide produced would explain ~89% (replicate 1) and ~80% (replicate 2) of the DIC produced. The lack of DIC production (11-20%) might be explained by biomass formation. We hypothesize that part of the hexadecane is incorporated into biomass and, therefore, not completely oxidized to CO_2. Overall, our results show sulfate reduction derived from ^{13}C-hexadecane oxidation and a DIC concentration matching the observed sulfide concentration.

Using the isotope data, we calculated that only 0.3 mM DIC derived from the labeled hexadecane (replicate 1) and 1.23 mM DIC (replicate 2) (Figure S4B), which explains only ~13% (replicate 1) and ~42% (replicate 2) of the DIC production. Our DIC calculations from $\delta^{13}C$ were substantially lower total DIC production. We attribute these results to an inefficient mixing of the non-labeled hexadecane. Furthermore, isotope fractionation (i.e., predominant oxidation of the non-labeled compounds) further reduce the observed shift in the labeled compounds.

Overall, our results show a good mass balance for the coupling of sulfate reduction and DIC formation in the *Ca.* Cerberiarchaeum culture, but only a fraction of the rate can be directly attributed to the turnover of ^{13}C-hexadecane.

SUPPLEMENTARY FIGURES

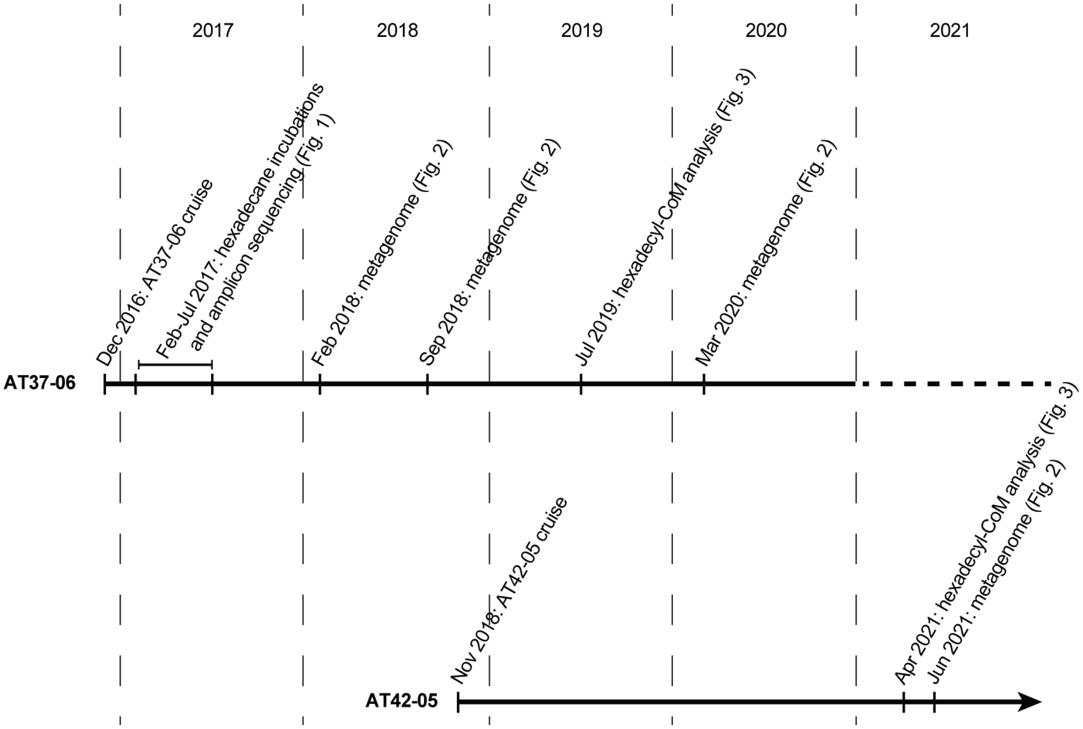

Figure S1. Cultivation, sequencing and metabolite analysis scheme from sampling campaigns AT37-06 and AT42-05.

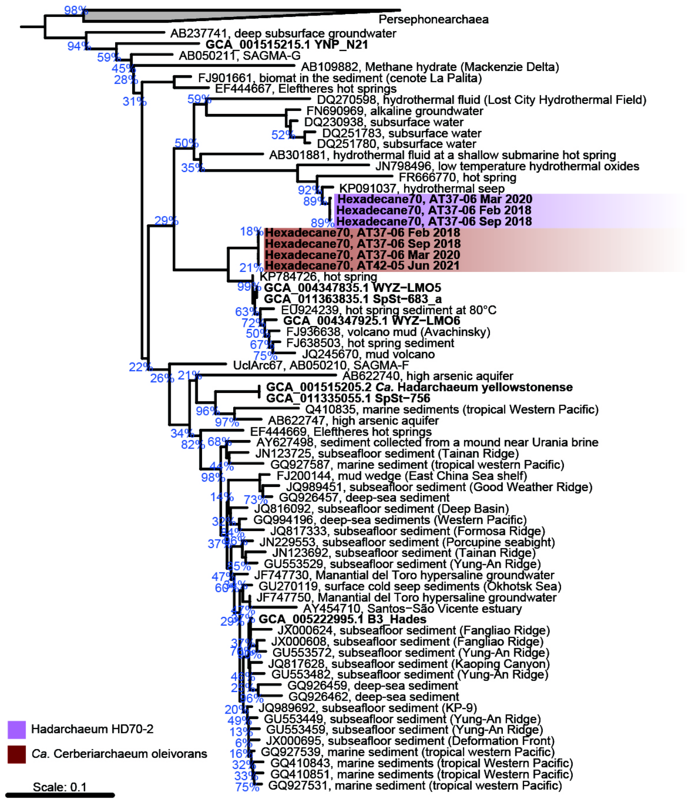

Figure S2. Placement of Hadarchaeota 16S rRNA genes in 16S rRNA gene phylogeny. Subset of 16S rRNA gene phylogeny of Archaea showing the Hadarchaea and Persephonearchaea clades. Hadarchaeota sequences from 4 metagenomic samples of the hexadecane70 enrichment are shown in bold and highlighted in red squares. 16S rRNA genes of Hadarchaeota MAGs are shown in bold. Blue numbers at the branches indicate bootstrap support. 300 archaeal sequences from the SILVA database [2] were aligned with the SINA alignment tool [3]. Maximum likelihood trees with 300 iterations were calculated with RAxML v. 7 [4] and the tree was visualized with the ARB software [1].

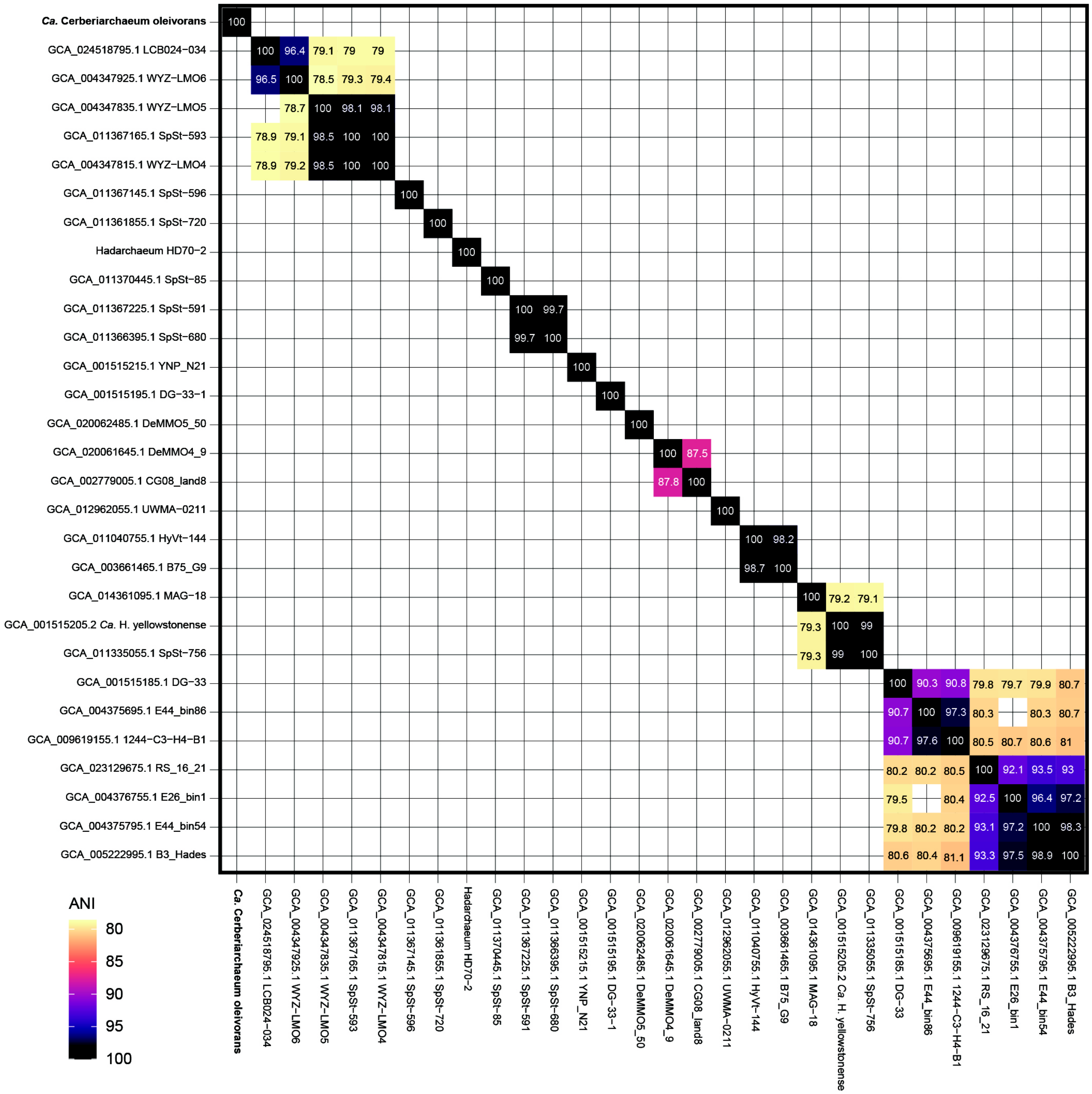

Figure S3. Average nucleotide identity between *Hadarchaeota* MAGs. ANI was calculated with FastANI [5]. Values below 75% are not shown.

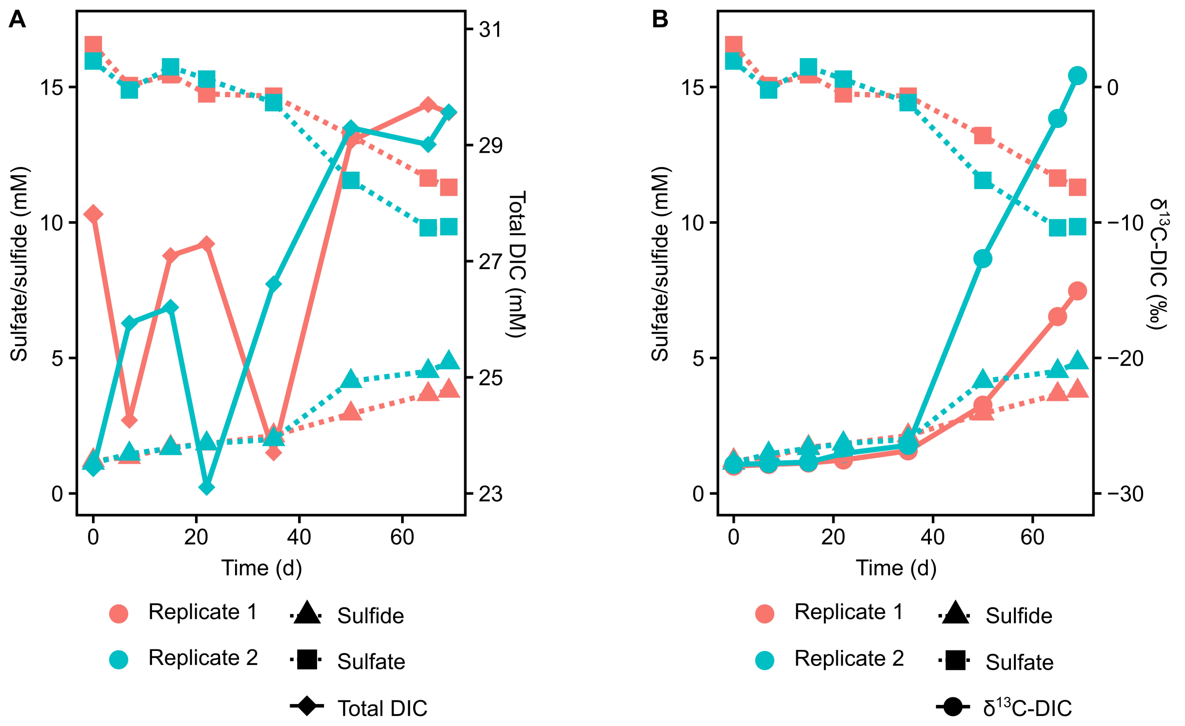

Figure S4. DIC production from ^{13}C-labeled hexadecane, sulfide production and sulfate consumption. A. DIC, sulfate and sulfide concentrations in two replicate experiments. The decrease in sulfate concentration corresponded to sulfide production and DIC formation. **B.** Incubation of two culture aliquots with ^{13}C-1,2-hexadecane produced substantial $^{13}CO_2$ (shown as increase in δ^{13}C values) after 35 days of incubation. Decrease in sulfate concentration corresponded to sulfide production.

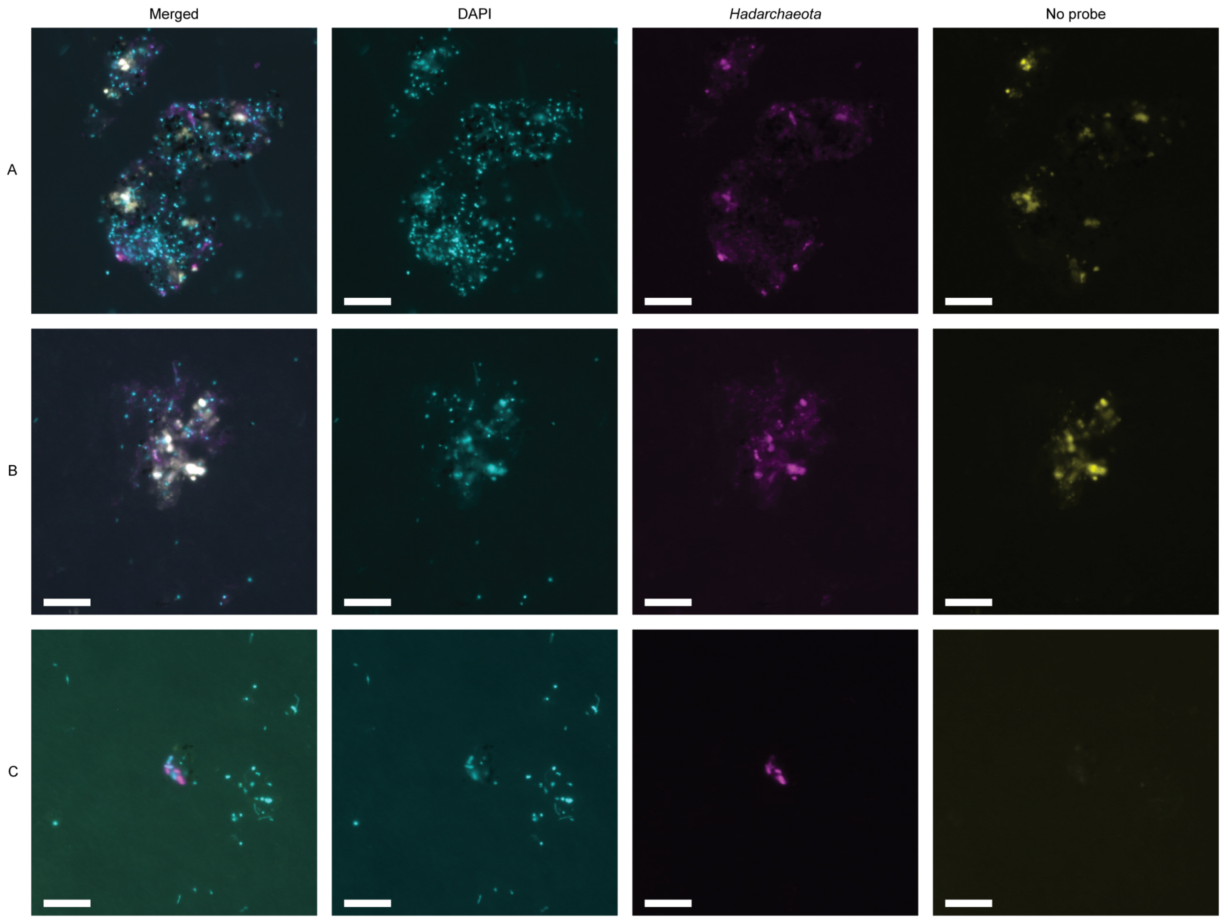

Figure S5. Visualization of *Hadarchaeota* cells in microbial aggregates. *Hadarchaeota* cells were visualized using a specific probe (Hades302, Table S1). A, B and C show three representative aggregates within the same preparation. The first column is the merged image of the three fluorescence channels. The second column shows the DNA stained in the aggregates (DAPI in cyan). The third column shows Hadarchaeota cells (Hades302 in purple). The fourth column shows background fluorescence (no probe, in yellow).

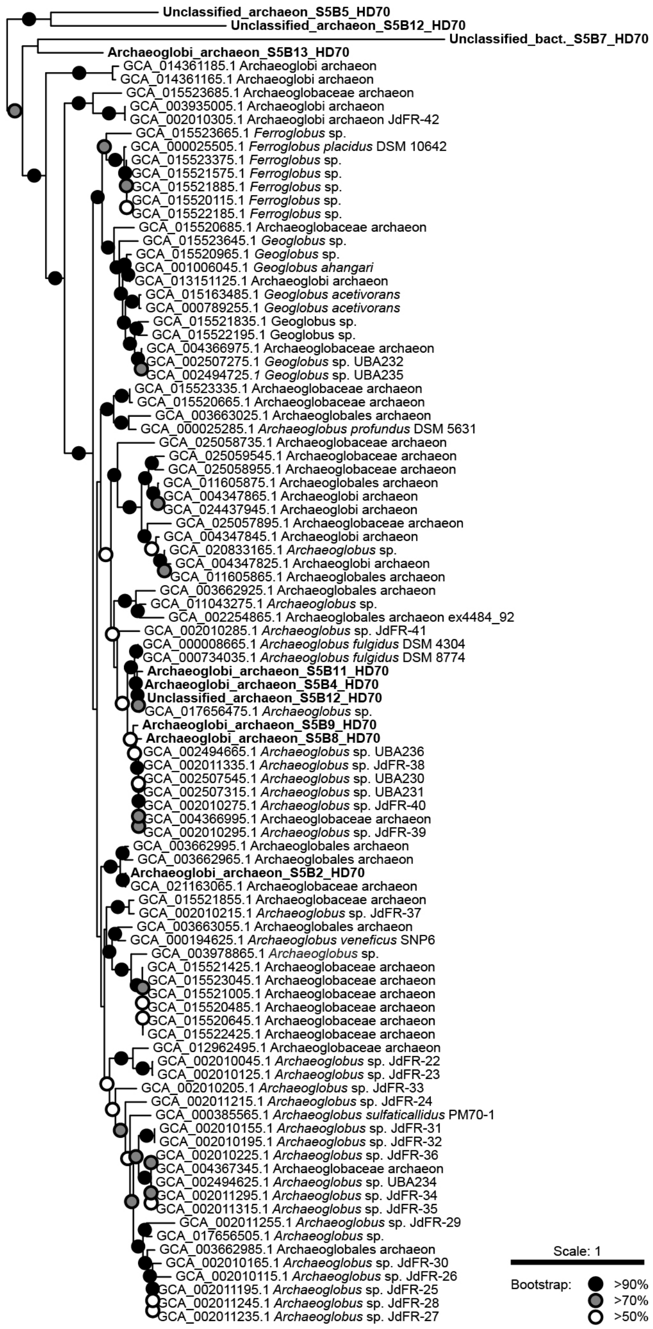

Figure S6. Placement of *Archaeoglobi* MAGs in phylogenomic species tree. Subset of species genome tree, showing *Archaeoglobi* and related MAGs. 38 archaeal marker proteins [6] were aligned with muscle [7] and a concatenated alignment was generated in anvi'o v.6 [8]. The maximum likelihood tree was calculated in IQTree using 100 bootstraps and the –test option to estimate the best substitution model for each protein [9].

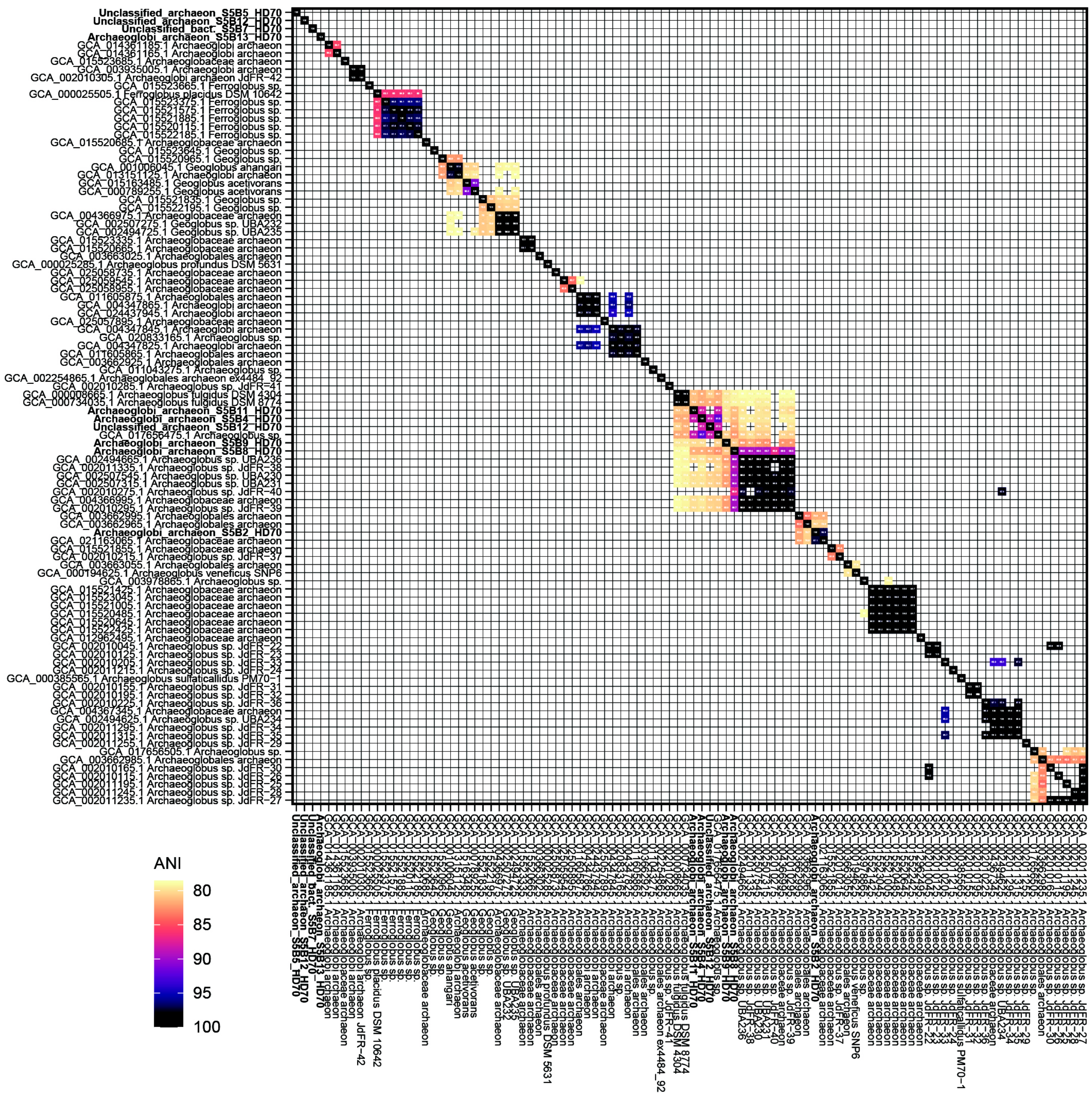

Figure S7. Average nucleotide identity between *Archaeoglobi* MAGs. The MAGs appear in the same order as in Figure S4. ANI was calculated with FastANI [5]. Values below 75% are not shown.

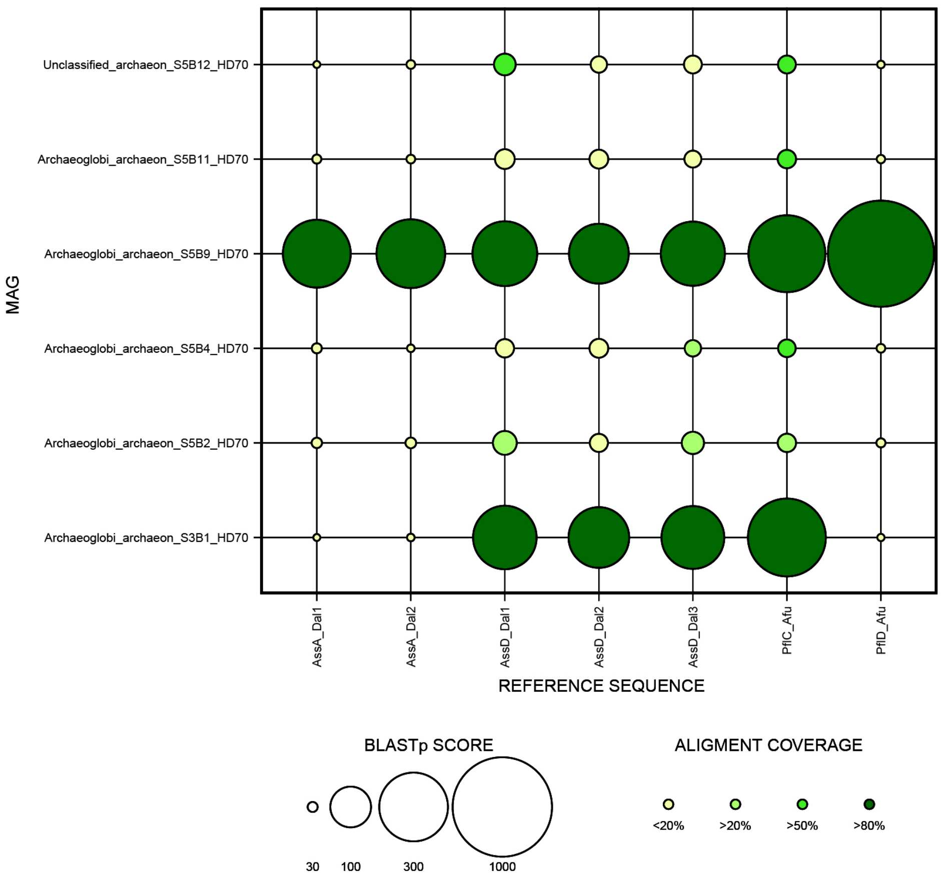

129 **Figure S8. BLASTp search of Ass/Pfl genes in *Archaeoglobi* MAGs.** The AssA and AssD of *Desulfatibacillum alkenivorans* and the PflC and PflD of *Archaeoglobus fulgidus* were queried against the proteins of *Archaeoglobi* MAGs. Darker colors represent longer sequence alignments and circle size represents higher BLASTp scores.

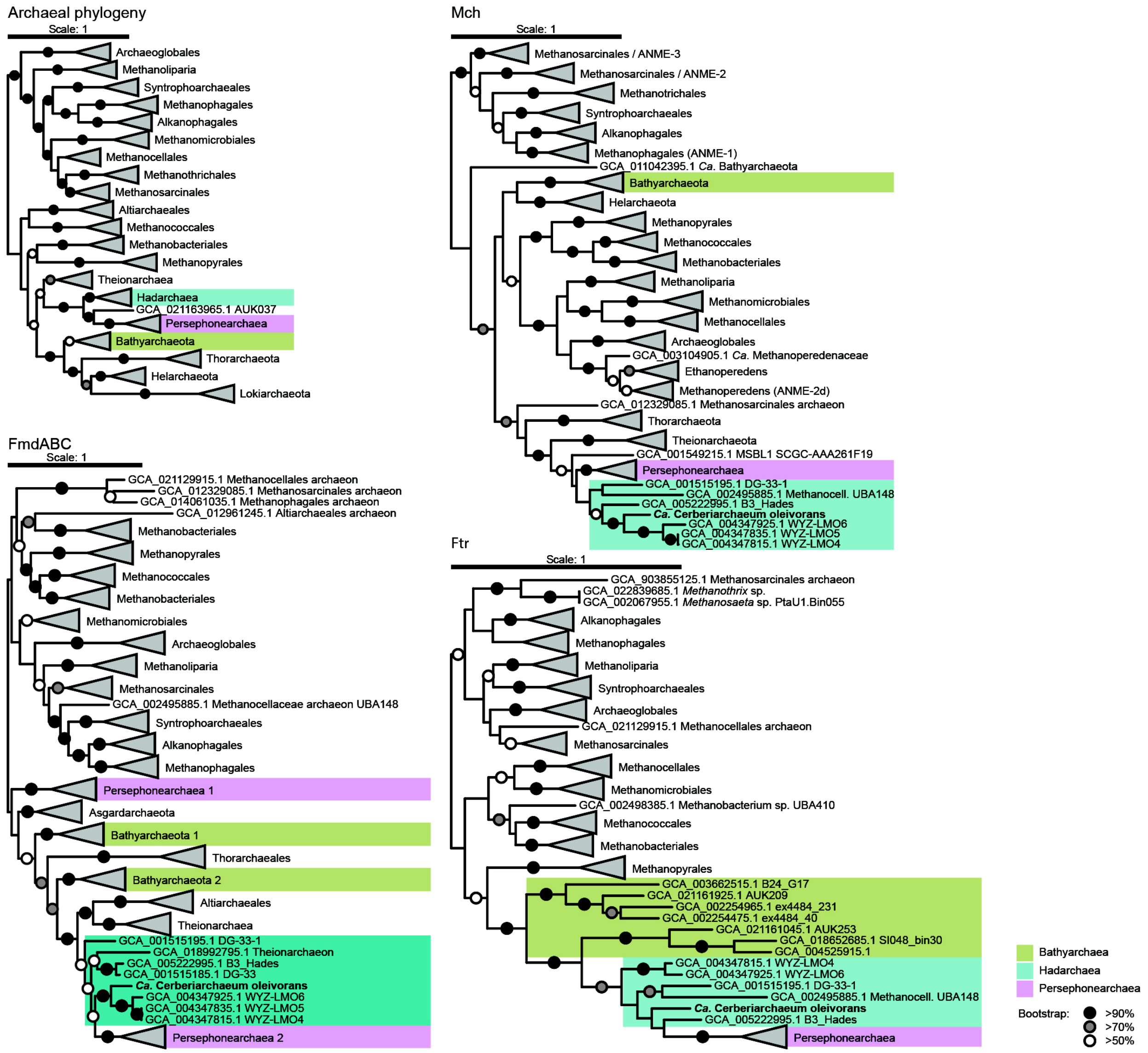

Figure S9. Placement of Hadarchaea in archaeal phylogenomic tree and in Mch, Fwd and Ftr phylogenies. Archaeal marker proteins and FwdABC phylogenies were done as in Figure S4. For Mch and Ftr, proteins predicted from PFAM models [10] were aligned with muscle [7]. Maximum likelihood trees with 100 bootstraps were calculated with IQTree [9].

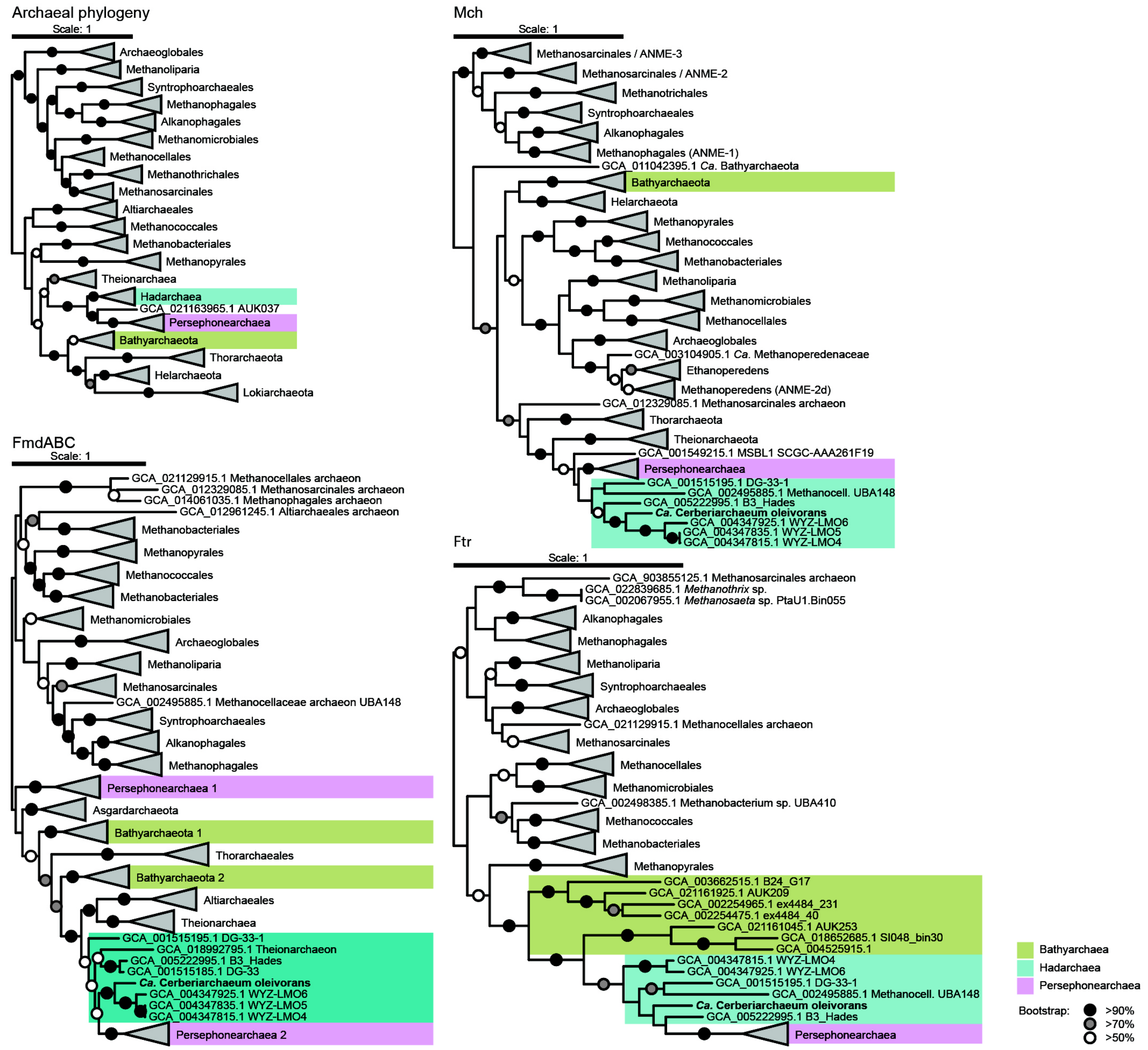

Figure S10. Phylogenies of formylmethanofuran dehydrogenase subunits A, B and C. The Fwd proteins were predicted using PFAMs [10]. Single proteins were aligned with muscle [7] and the trees were calculated in IQTree with 100 bootstraps [9].

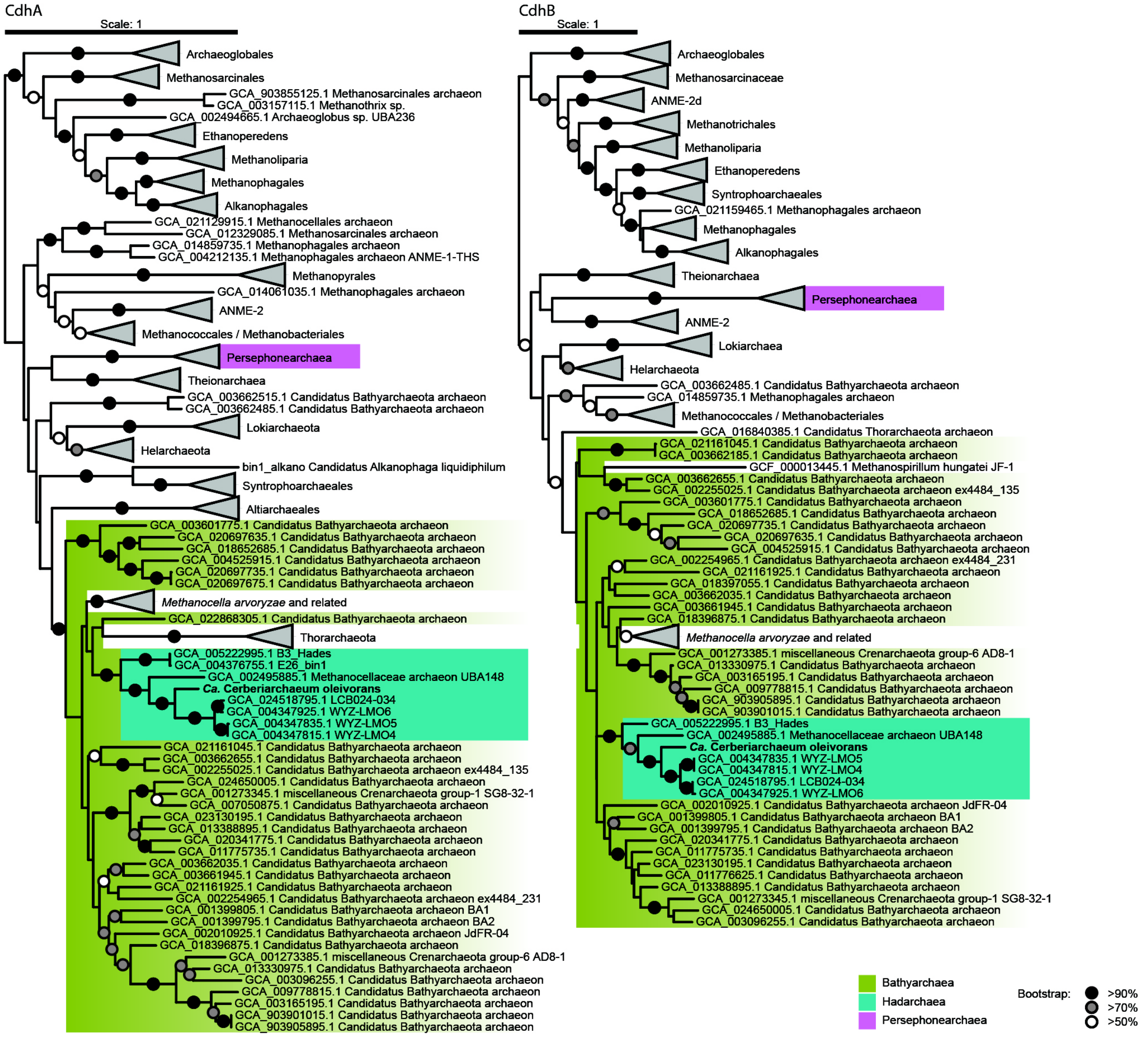

Figure S11. Phylogenies of Cdh subunits A, B, C, D and E. The Cdh proteins were predicted using custom hidden Markov models [11]. Single proteins were aligned with muscle [7] and the trees were calculated in IQTree with 100 bootstraps [9].

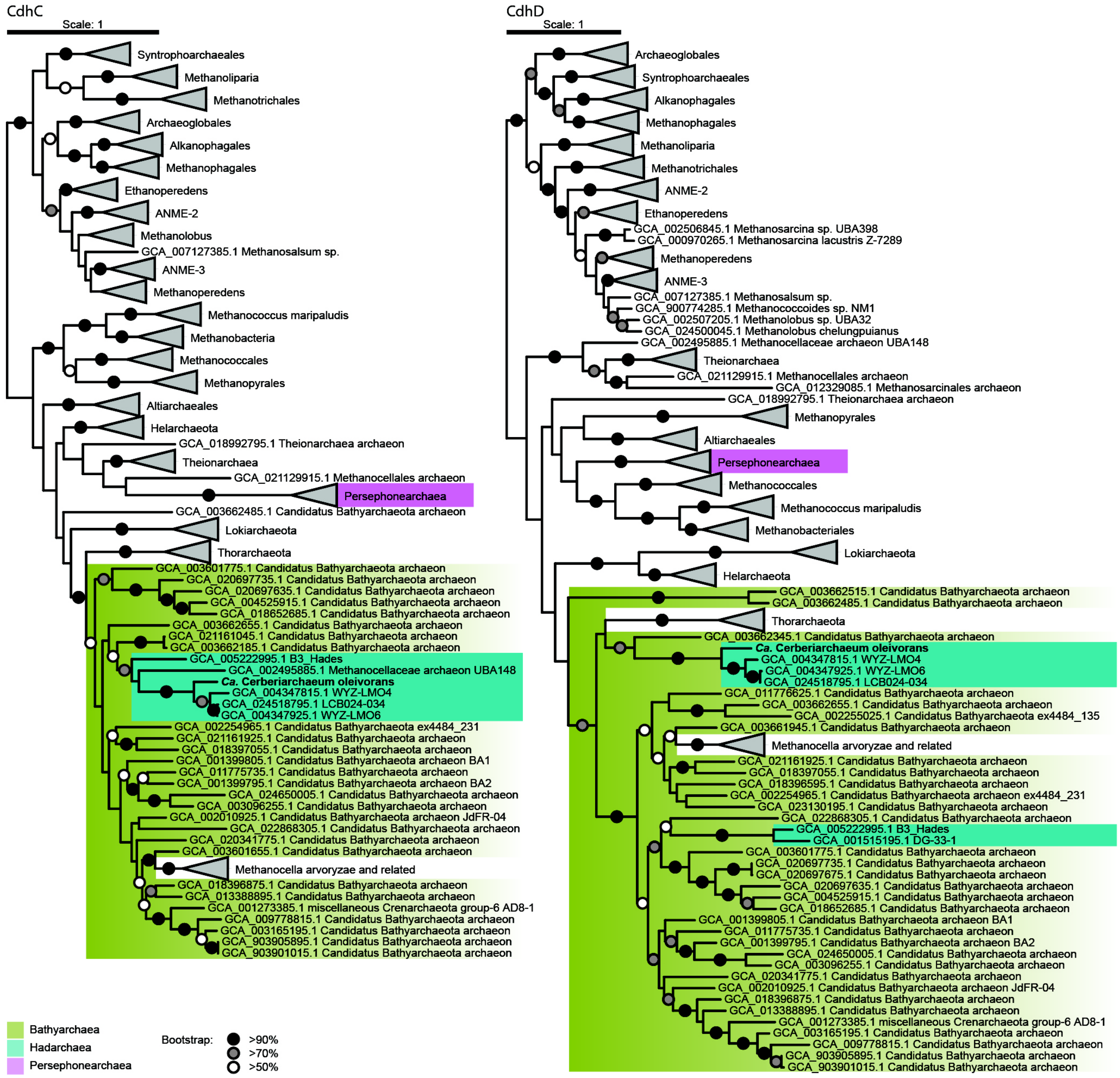

Figure S11 (continued). Phylogenies of Cdh subunits A, B, C, D and E.

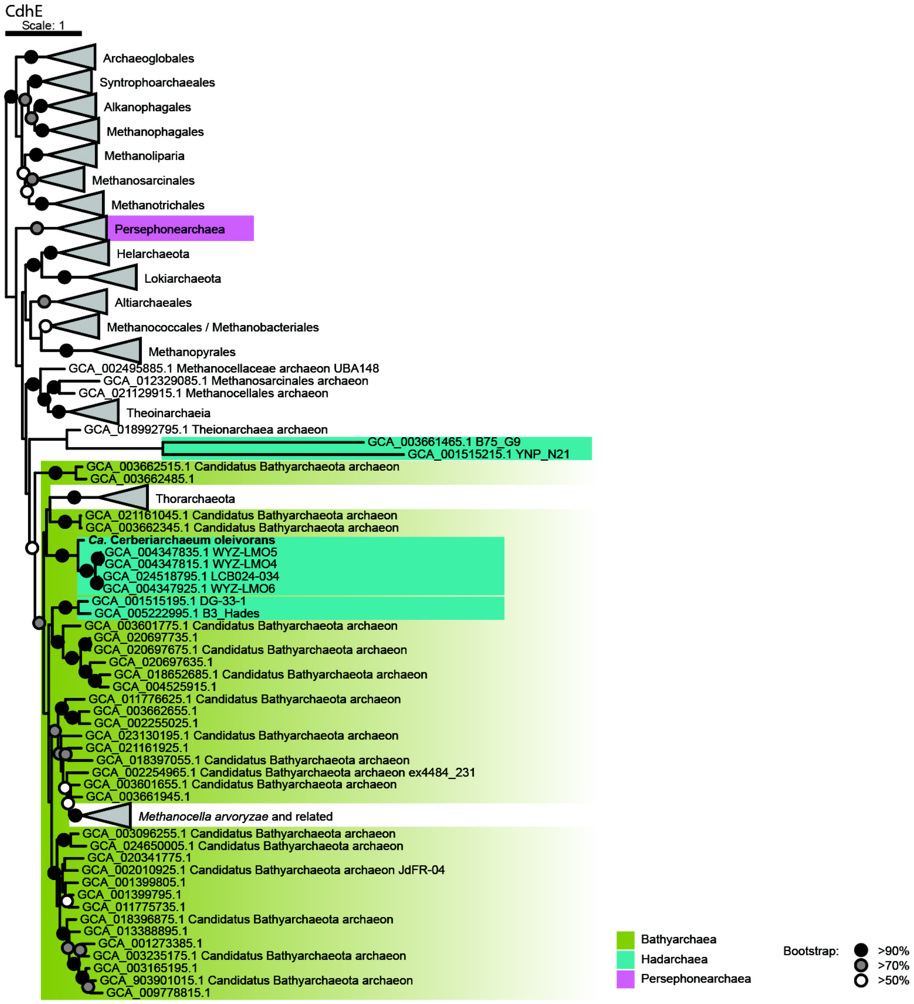

Figure S11 (continued). Phylogenies of Cdh subunits A, B, C, D and E.

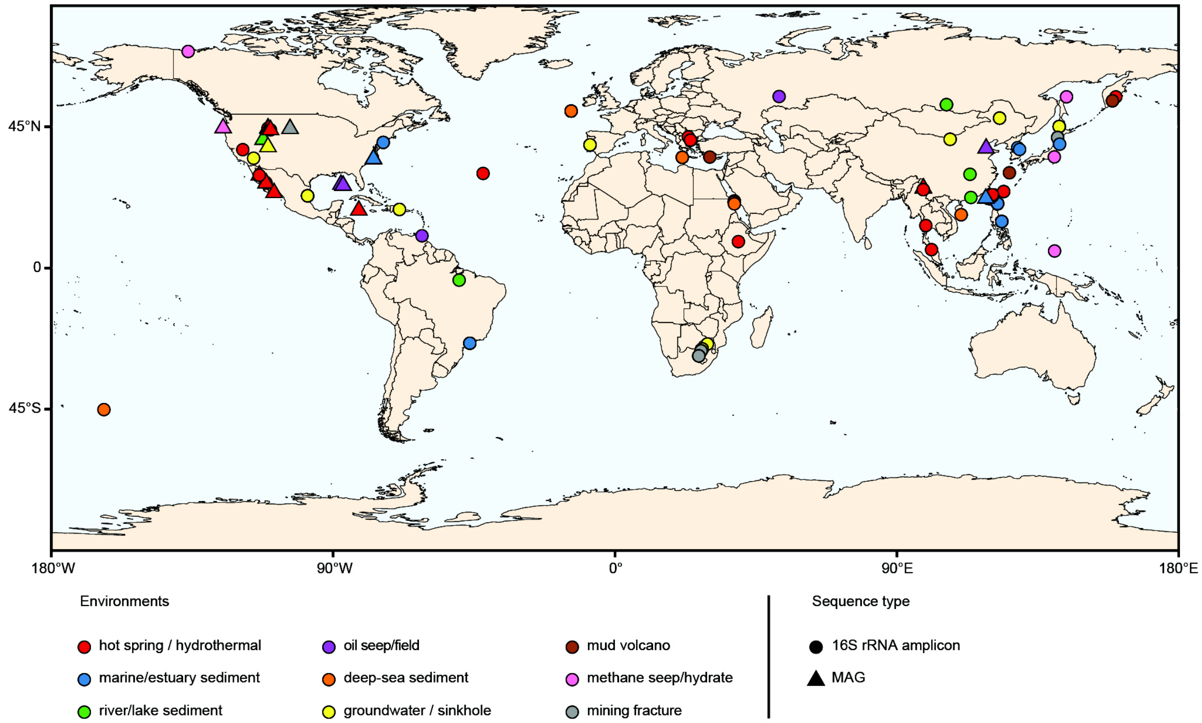

Figure S12. Global distribution of *Hadarchaeota* MAGs and 16S rRNA sequences. MAGs (triangles) and 16S rRNA sequences (circles) were retrieved from NCBI. Hadarchaea are globally distributed in anoxic subsurface environments and often associated with hydrothermal and alkane-rich environments.

SUPPLEMENTARY TABLES

Table S1. Primers used in 16S rRNA gene amplicon analysis and probes used in CARD-FISH experiments (see attached spreadsheet).

Table S2. Genomes used in phylogenomics of *Archaea* and archaeal single copy marker genes (see attached spreadsheet).

Table S3. Metagenome-assembled genomes from hexadecane70 enrichment metagenomes (see attached spreadsheet).

Table S4. Best BLASTp hits and bin affiliation of Mcr/Acr, Dsr and Ass genes found in metagenomic contigs (see attached spreadsheet).

Table S5. Annotation of MAGs described in the text (see attached spreadsheet).

Table S6. Best BLASTp hit of *Candidatus* Cerberiarchaeum oleivorans β-oxidation genes (see attached spreadsheet).

Table S7. Carbon metabolisms and optimal growth temperatures predicted for Hadarchaeota MAGs.

REFERENCES

1. Ludwig W, Strunk O, Westram R, Richter L, Meier H, Yadhukumar A, et al. ARB: a software environment for sequence data. *Nucleic Acids Res* 2004; **32**: 1363–1371.

2. Quast C, Pruesse E, Yilmaz P, Gerken J, Schweer T, Yarza P, et al. The SILVA ribosomal RNA gene database project: improved data processing and web-based tools. *Nucleic Acids Res* 2013; **41**: D590–D596.

3. Pruesse E, Peplies J, Glöckner FO. SINA: Accurate high-throughput multiple sequence alignment of ribosomal RNA genes. *Bioinformatics* 2012; **28**: 1823–1829.

4. Stamatakis A. RAxML version 8: a tool for phylogenetic analysis and post-analysis of large phylogenies. *Bioinformatics* 2014; **30**: 1312–1313.

5. Jain C, Rodriguez-R LM, Phillippy AM, Konstantinidis KT, Aluru S. High throughput ANI analysis of 90K prokaryotic genomes reveals clear species boundaries. *Nat Commun* 2018; **9**: 5114.

6. Darling AE, Jospin G, Lowe E, Matsen FA, Bik HM, Eisen JA. PhyloSift: phylogenetic analysis of genomes and metagenomes. *PeerJ* 2014; **2**: e243.

7. Edgar RC. MUSCLE: A multiple sequence alignment method with reduced time and space complexity. *BMC Bioinformatics* 2004; **5**: 113.

8. Eren AM, Kiefl E, Shaiber A, Veseli I, Miller SE, Schechter MS, et al. Community-led, integrated, reproducible multi-omics with anvi'o. *Nat Microbiol* 2020; **6**: 3–6.

9. Minh BQ, Schmidt HA, Chernomor O, Schrempf D, Woodhams MD, Von Haeseler A, et al. IQ-TREE 2: new models and efficient methods for phylogenetic inference in the genomic era. *Mol Biol Evol* 2020; **37**: 1530–1534.

10. Mistry J, Chuguransky S, Williams L, Qureshi M, Salazar GA, Sonnhammer ELL, et al. Pfam: The protein families database in 2021. *Nucleic Acids Res* 2021; **49**: D412–D419.

11. Adam PS, Borrel G, Gribaldo S. Evolutionary history of carbon monoxide dehydrogenase/acetyl-CoA synthase, one of the oldest enzymatic complexes. *Proc Natl Acad Sci* 2018; **115**: E1166–E1173.
